# Hypoxic preconditioned mesenchymal stem cells ameliorate rat brain injury after cardiopulmonary resuscitation by suppressing neuronal pyroptosis

**DOI:** 10.1111/jcmm.17782

**Published:** 2023-05-29

**Authors:** Xiahong Tang, Jun Ke, Falu Chen, Qingming Lin, Yan You, Nan Zheng, Zheng Gong, Xu Han, Yangping Zhuang, Feng Chen

**Affiliations:** ^1^ Shengli Clinical Medical College of Fujian Medical University Fujian Medical University Fuzhou China; ^2^ Department of Emergency Fujian Provincial Hospital Fuzhou China; ^3^ Fujian Provincial Key Laboratory of Emergency Medicine Fuzhou China; ^4^ The Second Department of Intensive Care Unit Fujian Provincial Hospital South Branch Fuzhou China

**Keywords:** bone marrow‐derived mesenchymal stem cells, cardiopulmonary resuscitation, HMGB1/TLR4/NF‐κB, hypoxic preconditioning, MAPK, pyroptosis

## Abstract

Cardiac arrest (CA) can result in cerebral ischaemia–reperfusion injury and poor neurological outcomes. While bone marrow‐derived mesenchymal stem cells (BMSCs) have been shown to have protective effects in brain ischaemic disease, their efficacy can be reduced by the poor oxygen environment. In this study, we investigated the neuroprotective effects of hypoxic preconditioned BMSCs (HP‐BMSCs) and normoxic BMSCs (N‐BMSCs) in a cardiac arrest rat model by examining their ability to ameliorate cell pyroptosis. The mechanism underlying the process was also explored. Cardiac arrest was induced in rats for 8 min and surviving rats received 1 × 10^6^ normoxic/hypoxic BMSCs or PBS via intracerebroventricular (ICV) transplantation. Neurological function of rats was evaluated using neurological deficit scores (NDSs) and examined for brain pathology. Serum S100B and neuron‐specific enolase (NSE) levels and cortical proinflammatory cytokines were measured to evaluate brain injury. Pyroptosis‐related proteins in the cortex after cardiopulmonary resuscitation (CPR) were measured using western blotting and immunofluorescent staining. Transplanted BMSCs were tracked using bioluminescence imaging. Results showed significantly better neurological function and neuropathological damage after transplantation with HP‐BMSCs. In addition, HP‐BMSCs reduced levels of pyroptosis‐related proteins in the rat cortex after CPR and significantly reduced levels of biomarkers for brain injury. Mechanistically, HP‐BMSCs alleviated brain injury by reducing the expressions of HMGB1, TLR4, NF‐κB p65, p38 MAPK and JNK in the cortex. Our study demonstrated that hypoxic preconditioning could enhance the efficacy of BMSCs in alleviating post‐resuscitation cortical pyroptosis. This effect may be related to the regulation of the HMGB1/TLR4/NF‐κB, MAPK signalling pathways.

## 1 INTRODUCTION

Cardiac arrest (CA) is a serious public health emergency with high morbidity and mortality rates.^1^, ^2^ Although the treatment of CA has improved, only 20%–40% of victims regain spontaneous circulation (ROSC).^3^ Brain damage is responsible for most deaths after ROSC.^1^ ROSC, followed by post‐CA syndrome, is characterized by global cerebral ischemia/reperfusion (I/R)‐induced injury, which contributes to a poor prognosis.^4^ Since the brain is highly susceptible to I/R injury, brain damage after cardiopulmonary resuscitation CPR accounts for two‐thirds of deaths in patients resuscitated out‐of‐hospital CA and nearly a quarter of patients who survive in‐hospital CA.^5^ Approximately 30% of survivors suffer from permanent injury brain, and half of the resuscitated patients are discharged from the hospital with varying degrees of neurological deficits.^6^, ^7^ Therefore, it is essential to explore the mechanisms underlying post‐resuscitation brain damage and identify neuroprotective drugs for resuscitated patients.

The mechanism underlying the increased brain damage after CPR resulting from global cerebral I/R remains unclear. However, inflammation is thought to play a significant role in secondary damage in brain ischaemic diseases.^8^ A growing body of evidence suggests that neuroinflammation triggered by brain I/R activates programmed cell death (PCD) signalling pathways within hours to days.^9^, ^10^ Numerous studies have indicated that regulating the apoptosis, necrosis, autophagy and pyroptosis pathways can achieve neuroprotection.^11^, ^12^, ^13^ Pyroptosis, a regulated cell death, has been observed in CPR models and is characterized by cell swelling and plasma‐membrane breakage.^13^ In addition, two recent studies have suggested that cell pyroptosis is extensively involved after CPR and could become a novel target for treating CPR.^13^, ^14^

Brain I/R damage generally activates the nod‐like receptor family protein 3 (NLRP3) inflammasome and secretion of pro‐inflammatory cytokines, including Interleukin‐1β (IL‐1β) and Interleukin‐18 (IL‐18).^15^ The NLRP3 inflammasome, including NLRP3, apoptosis‐associated speck‐like protein containing a CARD (ASC), and precursor of caspase‐1 (pro‐caspase‐1), plays an essential role in the initial phase of pyroptosis.^16^, ^17^ Subsequently, the activated caspase‐1 is cleaved, then processed by the inactive pro‐IL‐1βand pro‐IL‐18 and a protein named gadermin D (GSDMD), resulting in the formation of membrane pores and the release of abundant IL‐1β and IL‐18, causing cell pyroptosis.^18^, ^19^ Studies have confirmed that NLRP3 inflammasome‐dependent cell pyroptosis is involved in the pathogenic mechanism of whole brain I/R injury after CA/CPR.^13^, ^20^

Meanwhile, the high‐mobility family box‐1 (HMGB1) is also secreted extracellularly. HMGB1 binds to its receptors, toll‐like receptor 4 (TLR4) and RAGE, and regulates the expression of nuclear factor‐kB (NF‐κB), which expands the inflammatory response.^21^ The mitogen‐activated protein kinase (MAPK) is also a critical signalling pathway in the expression of NLRP3.^22^ Recent studies have demonstrated that suppressing pyroptosis can alleviate neuronal damage and improve neuropathy outcomes after resuscitation.^23^ However, research on neural function and pyroptosis after CA is still insufficient.

Mesenchymal stem cells (MSCs) have great potential in treating inflammation‐related diseases. Recent research has shown that MSCs harvested from various tissues, such as bone marrow, adipose tissue, umbilical cord blood and dental pulp, possess the ability to home inflamed tissue.^24^, ^25^, ^26^ Among these, bone marrow‐derived MSCs (BMSCs) are easy to obtain, have low antigenicity and are nearly non‐cytotoxic or oncogenic, making them a promising therapy against neurological disorders.^27^, ^28^ BMSCs release abundant neurotrophic factors and cytokines and activate signalling pathways to repair the I/R‐induced cell death, which potentially reduces brain damage and promotes nervous system recovery.^29^, ^30^, ^31^ Previous studies have found that transplantation with BMSCs relieves brain pathology and neurofunctional disturbance in CA/CPR rat model.^32^, ^33^ Our recent study demonstrated that transplantation with BMSCs attenuates brain damage in a rat CA model by decreasing levels of inflammatory factors and increasing levels of anti‐inflammatory cytokines.^34^ Although few studies have examined the effect of BMSC‐based therapies on reducing neural pyroptosis, there is evidence that BMSCs transplantation can improve neurological function.^35^, ^36^, ^37^

However, recent studies have shown that transplanted cells can be limited by the global and regional tissue microenvironment, decreasing the efficacy of BMSCs.^38^, ^39^, ^40^ Therefore, improving the viability of transplanted cells in the injured brain is crucial. Hypoxic preconditioning of BMSCs effectively increases cell survival during cerebral ischaemia,^41^, ^42^ as it can improve the cell's capacity to tolerate ischaemic areas.^43^ In addition, BMSCs can maintain their undifferentiated state and show migratory behaviour towards brain lesions under hypoxic culture conditions.^44^, ^45^ These findings suggest that HP may be an effective preconditioning method before transplantation. However, previous studies have only focused on localized brain ischaemia, and there have been no studies on the protective effect of hypoxic preconditioned BMSCs (HP‐BMSCs) against CA and post‐resuscitation. Notably, in vivo bioluminescence imaging (BLI) can accurately track the survival and migration of transplanted normoxic/hypoxic preconditioned BMSCs in the ventricles of the rat brain in a spatiotemporal manner.

Therefore, our study investigated the effect of HP on the neuroprotective potential of BMSCs in treating whole cerebral I/R injury. We intracerebroventricularly injected BMSCs into CA/CPR rats. CA/CPR was induced by asphyxia, and HP‐BMSCs were cultured with oxygen–glucose deprivation (OGD)‐injured neurons. We hypothesized that HP improves the efficacy of BMSCs, alleviating neurological dysfunction and relieving post‐resuscitation brain injury by inhibiting NLRP3 inflammasome‐mediated pyroptosis. Through this study, we furthered the understanding of the role of neural cell pyroptosis and the therapeutic effects of BMSCs against CA. How HP improves the therapeutic effects of transplanted BMSCs was also investigated. The results of our study are expected to provide valuable insights into the potential of HP‐BMSCs therapy as a treatment for post‐resuscitation brain injury.

## 2 MATERIALS AND METHODS

**FIGURE 1:**
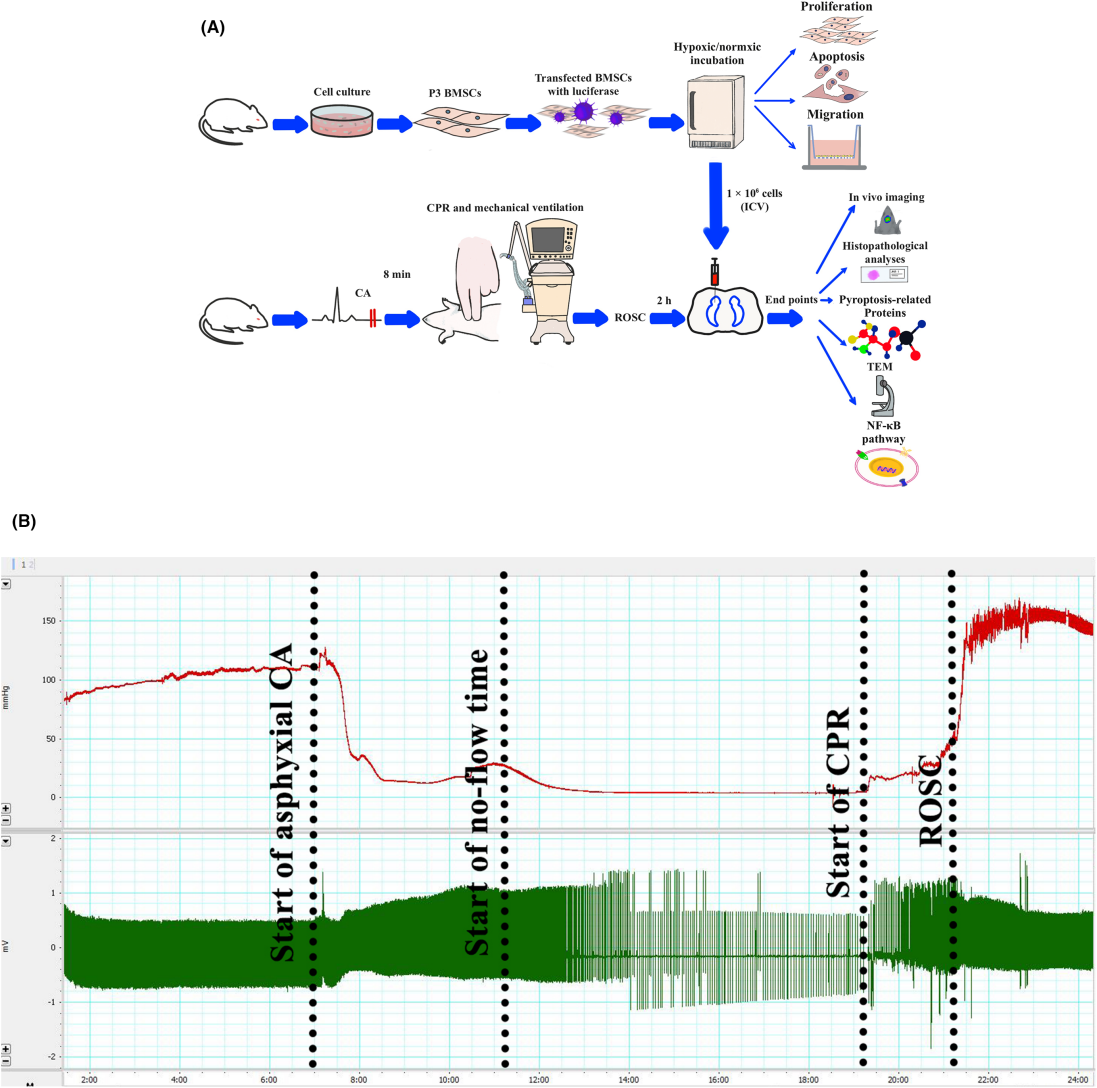
Experimental workflow and physiological surveillance data. (A) The schematic illustration represented the experimental plan. (B) Representative waveforms of femoral arterial pressure (red line) and electrocardiogram (green line) during the process of model establishment. CPR, cardiopulmonary resuscitation; ROSC, the return of spontaneous circulation (horizontal scaling = 1000:1).

Adult male SPF‐grade Sprague–Dawley (SD) rats (license no. SCXK (jing) 2019‐0008) were purchased from Beijing Huafukang Biotechnology Inc. and were housed under controlled temperature (22–25°C), humidity (60%–80% relative humidity) and illumination cycles (12 h/12 h light/dark cycles). BMSCs were isolated from five 4‐week‐old rats (90–100 g), whereas CPR was induced in eighty‐seven 8‐week‐old rats (200–250 g). In addition, cortical neurons were isolated from three SD pregnant rats at embryonic day 18. The rats received humane care in compliance with the NIH guidelines. All The experimental procedures for BMSC therapy are shown in Figure 1A. The protocol for this study was approved by the Animal Welfare and Ethics Committee of Fujian Medical University (license no. IACUC FJMU 2022‐0577).

The rats were randomly allocated into the following five groups: sham group (*n* = 15): rats underwent similar surgery without CA/CPR; CPR group (*n* = 18); rats underwent 8 min of asphyxial cardiac arrest and received CPR without treatment; CPR + PBS group (*n* = 18), in which rats received 15 μL PBS via lateral ventricle 2 h after ROSC; CPR + normal‐cultured BMSCs (N‐BMSCs) group (*n* = 18), in which BMSCs were injected into lateral ventricle 2 h after ROSC and CPR + HP‐BMSCs group (*n* = 18), in which the lateral ventricle was injected with HP‐BMSCs 2 h after resuscitation. The five groups were divided into three subgroups: 6, 12 and 24 h, with six test rats in each subgroup, and each sham subgroup had five rats. All efforts were made to minimize the consumption and pain of animals in the study.

### 2.1 Isolation and characterization of BMSCs

Isolation and culture of BMSCs were performed according to a previously published protocol but with minor modifications.^46^ Briefly, young rats were rapidly anaesthetized with sevoflurane and sacrificed by dislocating cervical vertebrae. BMSCs were collected from the femur and tibia by flushing the medullary canal with Dulbecco's Modified Eagle Medium (DMEM)/F12 (Gibco). The medium was centrifuged at 1500 rpm for 5 min to isolate the BMSCs. The cells were resuspended in 4 mL DMEM/F12 containing 10% foetal bovine serum (Clark) and cultured in 25 cm^2^ culture flasks in an incubator (Sanyo) at 37°C and 5% CO_2_. The cells were detached with 0.25% trypsin‐ethylenediaminetetraacetic acid (EDTA) (Gibco) when they reached 90% confluence. Passage 3 (P3) cells were identified using flow cytometry (BD Bioscience) as described previously,^34^ using the following antibodies: anti‐CD29‐FITC, CD45‐FITC, anti‐CD90‐PE and anti‐CD11b‐PE (all from BioLegend). Well‐growing cells at passages 3–8 were used in subsequent experiments.

### 2.2 Preparation of HP‐BMSCs

For hypoxic culture, BMSCs have transferred to a hypoxic incubator (Biospherix) incubator and incubated at 37°C, 5% CO_2_, 3% O_2_ and 92% N_2_ for 48 h.

### 2.3 Transfection of BMSCs with luciferase lentiviral vectors

Before transplantation or hypoxic preconditioning, half of the BMSCs in each normoxic/hypoxic treated subgroup (6, 12 and 24 h, *n* = 6) were suspended in 3.6 mL medium and infected with 400 μL of 1 × 10^8^ transduction units (TU)/mL lentiviral vectors carrying luciferase (Genepharma). The cultures were maintained in 25 cm^2^ flasks at 37°C with 5% CO_2._ The other three cell samples were not labelled with lentivirus.

### 2.4 Determination of cell viability

Cell viability was determined using the Cell Counting Kit‐8 (CCK‐8, Meilun Biotech). Briefly, P3 cells were inoculated in 96‐well plates at a density of 2 × 10^3^ cells per well and incubated overnight. Cells were then maintained under normoxic conditions or exposed to hypoxia, as described above, for 48 h. Four hours before hypoxia exposure was terminated, CCK‐8 solution (10% in the growth medium, 100 μL/well) was added, and cells were incubated for an additional 4 h. Absorbance was measured at 450 nm using a microplate reader (BioRad).

### 2.5 Determination of cell apoptosis

The apoptosis of BMSCs was evaluated using an Annexin V‐FITC/propidium iodide (PI) apoptosis detection kit (Meilun Biotech). Briefly, cells were treated with or without HP and then dissociated and washed twice with PBS. The cell suspensions were incubated with 5 μL Annexin V‐FITC and 5 μL PI at room temperature for 15 min in the dark. The cells were analysed using a flow cytometer (BD), and the apoptosis was quantified using Accuri C6 Plus software (BD). All experiments were performed five times.

### 2.6 Transwell migration assay

Cell migration was evaluated using a 24‐well Transwell chamber with 8 μm filter inserts (Corning). Briefly, normoxic/hypoxic BMSCs (1 × 10^5^ cells) were plated in the upper layer with 200 μL serum‐free medium, and 1 mL of complete medium supplemented with 10% FBS was added to the lower compartment. Cells were cultured at 37°C, 5% CO_2_ for 24 h. Subsequently, a wet cotton swab was used to wipe off non‐migrated cells from the upper layer, and migrated cells on the lower surface were fixed in 4% paraformaldehyde for 15 min. The cells were then stained with 0.1% crystal violet (Solaibao) and observed under an inverted microscope (Leica). The migrated cells were counted using the ImageJ software (NIH). Three random fields were selected from each sample, and the average was calculated.

### 2.7 Establishment of CA/CPR model

The rats were starved overnight but with access to water. The rats were initially anaesthetized with sevoflurane (Abbvie) and then deeply anaesthetized with pentobarbital (45 mg/kg) by intraperitoneal injection. Anaesthesia was maintained by giving additional pentobarbital (10 mg/kg) as needed.

A 14‐gauge tracheal catheter (BD) was inserted through the orotracheal tube, and the cannula was connected to a small animal ventilator (Acott Biotech) with the following settings: ventilation frequency at 100 breaths/min; tidal volume of 6.5 mL/kg, and inhaled oxygen concentration of 21%. A polyethylene‐30 (PE‐30) tube (SDR Scientific) was inserted into the right femoral artery and connected to a pressure transducer for continuous monitoring of mean arterial pressure (MAP). The catheter was flushed with heparinized saline (2.5 IU/mL) when necessary. Heart rhythm was monitored by a standard lead II electrocardiogram. The MAP and electrocardiogram signals were recorded and digitized via the Power lab data acquisition system (AD Instrument) and continuously collected for later analysis.

According to our previous study,^34^ CA is caused by 6‐min asphyxia of less pronounced brain damage symptoms. Therefore, in the present study, CA time was extended to 8 min. The rat's temperature was measured through a rectal probe and maintained between 36.5°C and 37°C by a thermostat (RWD Life Science).

After establishing a stable baseline for 5 min, vecuronium (Xianju Pharmaceutical, 1 mg/kg) was administered through the right femoral artery, and asphyxia CA was induced by switching off the ventilator. CA was defined as MAP less than 25 mmHg in combination with pulseless electrical activity, ventricular fibrillation or cardiac arrest. The circulatory arrest induction was performed for 3–4 min (Figure 1B). Ventilation assistance was provided after 7 min 45 s of cardiac arrest with 100% O_2_, and the remaining parameters were unchanged. Chest compressions were initiated when CA lasted for 8 min, and manual chest compressions were maintained at a rate of 200 times per min. The compression depth was adjusted to maintain a MAP ≥20 mmHg. Epinephrine (0.05 mg/kg) and heparinized saline (0.5 IU in 0.1 mL) were administered via the femoral artery catheter 1 min after the start of CPR, ROSC was defined as MAP ≥60 mmHg, lasting for more than 5 min. If ROSC did not occur within 2 min, the CPR was stopped. The respiratory machine was kept on to provide 100% O_2_ for 1 h after ROSC. The inhaled oxygen concentration was reduced to 21% for 1 h. The endotracheal tube and catheter were removed, and the skin was sutured. Each rat received an intraperitoneal injection of 0.1 million units of penicillin to prevent infection after surgery. The operation was performed by the same operator to reduce experimental variations. All animals were operated under a sterile environment, and no infected wounds were observed. The rats were returned to their individual cages, and corncob was used for bedding. Rats meeting any of the following criteria were excluded from the study: (1) Rats that failed to achieve ROSC within 2 min of CPR initiation; (2) Rats that were difficult to wean from the ventilator 2 h after ROSC and (3) Rats that died prior to sampling.

### 2.8 Treatment processes

Two hours after resuscitation, the rats in the CPR + PBS group received a 15 μL intracerebroventricular injection of PBS, while rats in the CPR + N‐BMSCs group and CPR + HP‐BMSCs group received a single injection of 15 μL PBS containing 1 × 10^6^ N‐BMSCs or HP‐BMSCs to the left lateral ventricle. Pentobarbital was given if additional anaesthesia was necessary. The anaesthetised rats were secured in a stereoscopic frame (RWD Life Science) and a small incision was made to expose the skull. The skull position was adjusted until the bregma‐lambda axis was horizontal. A hole (coordinates: P—0.8 mm, L—1.5 mm, D—3.9 mm) was drilled into the skull using a mini‐drill (RWD Life Science). The infusions were administered at a rate of 1 μL/min using a 25 μL Hamilton syringe (Hamilton) that was connected to a microinjection pump (RWD Life Science). After injection, the needle remained in position for 15 min to prevent reflux. The holes were covered with medical bone wax (Johnson & Johnson), and the incision was sutured.

### 2.9 In vivo imaging of luciferase‐expressing BMSCs

The survival and distribution of the transplanted cells were analysed using an in vivo imaging system (IVIS, PerkinElmer). Rats were anaesthetised with pentobarbital and received D‐luciferin potassium salt (PerkinElmer, 150 mg/kg) intraperitoneally at 6, 12 and 24 h after ROSC. Images were captured 20 min after luciferin administration, and cell survival was observed in the brain ventricle. The luminescence of BMSCs was measured using Living Image software (PerkinElmer).

### 2.10 Specimen collection

Neurological outcomes were accessed at 6, 12 and 24 h after ROSC, while rats were under sevoflurane anaesthesia. The animals were sacrificed by cervical dislocation immediately after blood samples were obtained through heart puncture. The rats were then transcardially perfused with ice‐cold physiological saline to remove residual blood until no red perfusate was effused. The brains were quickly removed and placed on ice for dissection. The right frontal cortex of each rat was used for electron microscopy. The right parietal cortex was carefully collected for histopathological and immunofluorescence staining analysis. Meanwhile, the left frontal cortical tissues were homogenized, and the supernatants were used for enzyme‐linked immunosorbent assay (ELISA). The left parietal cortex was saved to detect the expressions of target proteins via western blotting. Operators involved in detection and data collection were blinded to the experimental treatments.

### 2.11 Neurological function assessment

Neurological function was evaluated at each time point according to a previously described protocol.^47^ The neurological deficit scores (NDS) were based on assessments of overall behaviour, brainstem function, motor function, sensory function, movement behaviour and signs of seizures. Scores ranged from 0 to 80, with a score of 80 indicating normal neurological function and a score of 0 indicating brain death. Lower scores indicated severe neurological deficits.

### 2.12 Histological evaluation of brain cerebral tissues

Brain tissues were fixed in 4% paraformaldehyde overnight, embedded in paraffin after dehydration with gradient alcohol, and cut into 5‐μm‐thick coronal sections. Sectioned slides were stained by haematoxylin–eosin according to the protocol and examined by light microscopy.

### 2.13 Immunofluorescence assay

The brain sections were first dewaxed and then underwent antigen retrieval by immersion in EDTA antigen repair buffer (PH 8.0) and heating. After washing with PBS (PH 7.4), sections were blocked in 3% bovine serum albumin (BSA, Servicebio) for 30 min, followed by the removal of BSA. Primary antibodies were added to the sections and incubated overnight at 4°C. The sections were then washed three times with PBS for 5 min each and incubated with fluorescein‐labelled secondary antibody (Servicebio) for 50 min in the dark. After washing three more times with PBS, the sections were stained with DAPI (Servicebio) for 10 min and sealed with an anti‐fluorescence quencher. Fluorescent microscope images (Leica) were captured to visualize DAPI (blue), NeuN (green), NLRP3 (red) and cleaved‐caspase‐1 (red).

### 2.14 ELISA

The levels of IL‐1β and IL‐18 in the left frontal cortex samples were measured using rat IL‐1β and IL‐18 ELISA kits (Cloud‐Clone) according to the manufacturer's instructions. In addition, the concentrations of serum S100B and neuron‐specific enolase (NSE) were measured using the rat S100B test kit (Cloud‐Clone) and the rat NSE reagent kit (Cloud‐Clone), respectively. Optical densities at 450 nm were measured using a microplate reader C (Rayto Life Science).

### 2.15 Western blotting

First, cortical tissues were washed three times with PBS to remove the blood and cut into small pieces. Next, tissue samples were lysed and centrifuged, and the resulting supernatant was collected as the total protein solution. Total protein concentration was determined using a bicinchoninic acid (BCA) protein assay kit (Beyotime). The proteins were then separated using sodium dodecyl sulfate‐polyacrylamide gel electrophoresis (SDS‐PAGE), followed by transfer to polyvinylidene difluoride (PVDF) membranes. The membranes were blocked with 5% non‐fat dry milk in Tris‐buffered saline containing Tween 20 (TBST) and probed with primary antibodies overnight at 4°C, including anti‐NLRP3 (Immunoway), ASC (Immunoway), cleaved‐caspase‐1 (Immunoway), GSDMD‐N (Immunoway, United Kingdom), HMGB1 (Bioss), TLR4 (Bioss), NF‐κB p65 (Immunoway), p38 MAPK (Immunoway), JNK (Immunoway) and anti‐β‐actin (CST) antibodies. After washing three times with TBST, the membranes were incubated with the corresponding secondary antibodies (Servicebio), and the protein bands were detected using a Bio‐Rad Gel imaging system (Bio‐Rad). The quantification of protein bands was performed using Image J software, and the Western blot results were normalized to β‐actin expression.

### 2.16 Electron microscopy examination

The right frontal cortical tissues were fixed in 2.5% glutaraldehyde in 0.1 M phosphate buffer (PH 7.4). The samples were then rinsed three times in 0.1 M phosphate buffer (PH 7.4) and further fixed in 1% osmic acid (Ted Pella) for 2 h. After another round of rinsing in 0.1 M phosphate buffer (PH 7.4), the samples were dehydrated in various concentrations of alcohol for 20 min and 100% acetone for 15 min twice. Following dehydration, the samples were embedded in resin and left to polymerize at 60°C for at least 48 h. Ultrathin sections (60–80 nm thick) were cut with a diamond knife (Daitome) on an ultra‐thin microtome (Leica). These sections were placed on 150 mesh copper grids and stained with 2% uranyl acetate saturated alcohol solution to avoid light staining for 8 min. Finally, the sections were stained with 2.6% lead citrate to avoid CO_2_ for 8 min and rinsed in ultrapure water three times. The high‐resolution transmission electron microscopy (TEM) images were acquired on a TEM instrument (Hitachi).

### 2.17 Primary cortical neuron culture

Primary cortical neurons were obtained from embryonic day 18 rats. The Cortical tissues were cut into small pieces with scissors and gently trypsinized with a 0.25% Trypsin–EDTA (Gibco) solution for 30 min. The resulting tissue suspensions were filtered through a 70‐μm cell strainer and resuspended in DMEM/F12 media (Gibco). The suspensions were seeded in plates coated with polylysine and cultured at 37°C. After 8 h, the medium was replaced with Neurobasal medium (Gibco) supplemented with 2 mM glutamine (Meilun), 2% B27 (Gibco) and 1% penicillin/streptomycin (Meilun). On the third day, 10 μM cytarabine (APExBIO) was added to inhibit glial proliferation. The mature neurons were identified by anti‐MAP2 (1:200, Bioss) staining (red). Cortical neurons that were cultured for 10 days were subjected to oxygen–glucose deprivation and reperfusion (OGD/R) or co‐cultured with BMSCs.

### 2.18 Establishment of the OGD/R model

The primary cultured neurons were washed twice with PBS and cultured in a glucose‐free DMEM medium (Gibco). Subsequently, the neurons were exposed to oxygen–glucose deprivation (OGD) in an anaerobic chamber (Biospherix) for 2 h, with a gas mixture of 95% N_2_ and 5% CO_2_. After the OGD period, the neurons were returned to their original medium and co‐cultured with normoxic/hypoxic BMSCs.

### 2.19 Co‐culture of injured neurons with BMSCs under different oxygen levels

For the co‐culture experiment, normoxic or hypoxic BMSCs were seeded on the upper layer of the Transwell chamber (Corning) at a density of 5 × 10^4^ cells/well and cultured for 4 days in a hypoxic chamber (3% O_2_, 5% CO_2_ and 92% N_2_). Next, the DMEM/F12 medium was replaced with a Neurobasal medium. Neurons that had undergone OGD were cultured in the lower layer of the Transwell with a Neurobasal medium. The cells were co‐cultured for 24 h at 37°C with 5% CO_2_. This part of the experiment involved seven groups (*n* = 3 per group): control group, OGD group, OGD + N‐BMSCs group, OGD + HP‐BMSCs group, OGD + HP‐BMSCs+SB203580 group (p38 MAPK inhibitor, 10 mM, APExBIO), OGD/R + HP‐BMSCs+ SP600125 group (JNK inhibitor, 10 mM, APExBIO) and OGD + HP‐BMSCs+ Bay11‐7082 group (NF‐κB inhibitor, 20 mM, APExBIO). Specific pathway inhibitors were added to the lower Transwell chamber simultaneously with co‐culture.

### 2.20 Statistical analysis

The Shapiro–Wilk normality test was used to test for normality. All experiments were replicated at least three times. Data that satisfied the normality conditions were presented as means ± standard deviations (SD). Statistical analysis and plotting were performed using SPSS 19.0 (IBM) and GraphPad Prism 9.0 software (GraphPad Prism Software). For normally distributed data, comparisons between two groups were analysed by Student's *t*‐test, while one‐way anova was used to compare data from more than two groups. In cases where the Brown‐Forsythe test showed data variances were not equal, the Brown‐Forsythe anova test was performed, followed by Dunnett's T3 multiple comparisons test. *p* < 0.05 were considered statistically significant.

## 3 RESULTS

### 3.1 The effect of hypoxic conditions on cell migration in BMSCs

**FIGURE 2:**
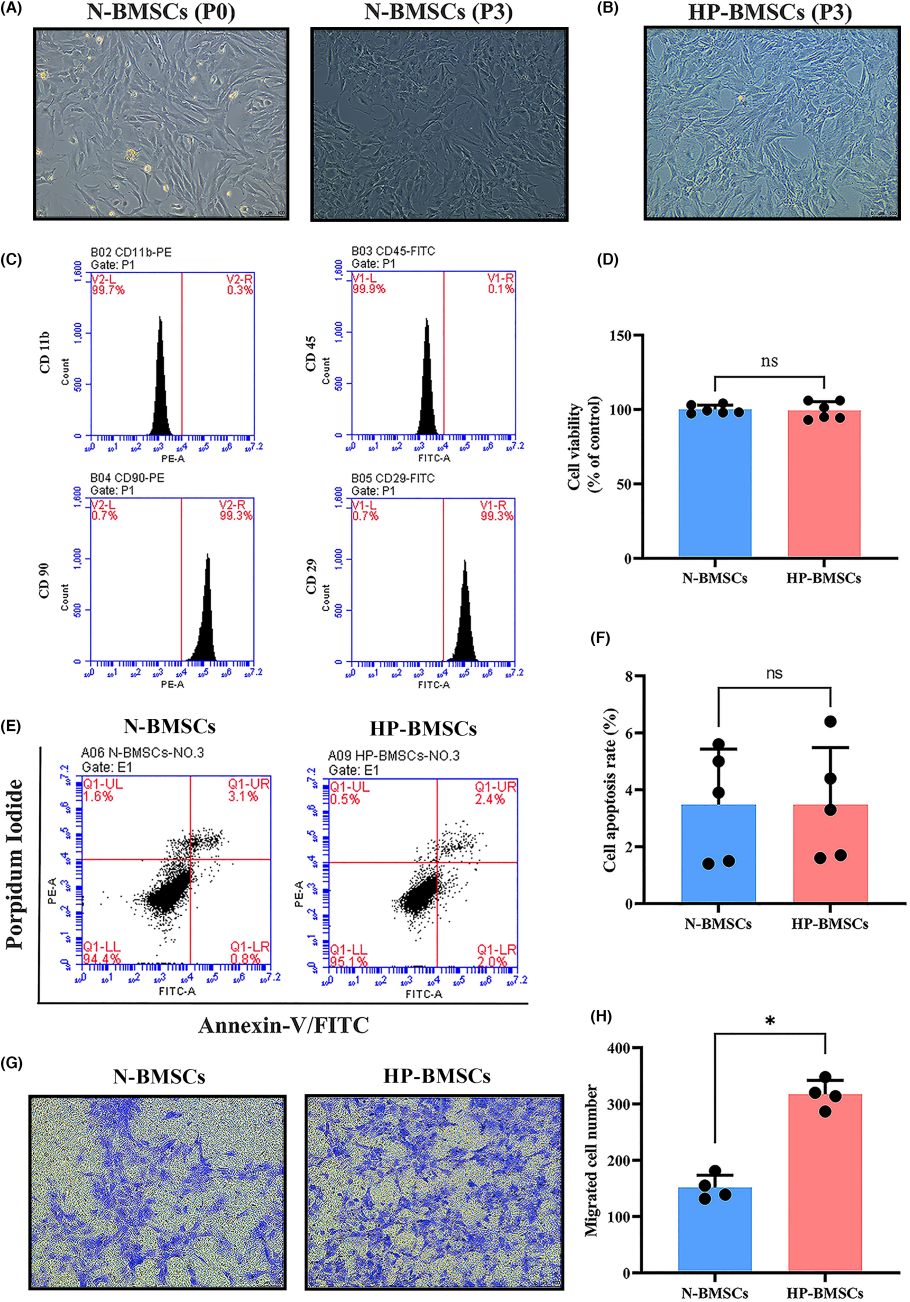
Characteristics of bone marrow‐derived mesenchymal stem cells (BMSCs) under normoxic or hypoxic conditions. (A) Representative images of passages 0 (P0) and 3 (P3). (B) Morphological appearance of hypoxic preconditioned BMSCs (HP‐BMSCs) at P3. Scale bar = 100 μm. (C) Flow cytometric results indicated that BMSCs at P3 were positive for CD29 and CD90 and negative for CD11b and CD 45. (D) Cell Counting Kit‐8 (CCK‐8) analysis revealed hypoxic conditions for 48 h in culture did not promote BMSCs proliferation compared with the normoxic cultured cells (21% O_2_, *n* = 6). (E, F) Annexin V/propidium iodide (PI) showed hypoxia for 48 h and did not induce apoptosis of BMSCs (*n* = 5). (G, H) Transwell assay demonstrated hypoxic preconditioning increased the BMSCs migration compared with the normal‐cultured BMSCs (N‐BMSCs, *n* = 4). Scale bar = 100 μm. All data are presented as means ± SD. *p < 0.05 vs. the N‐BMSCs group. Ns *p* > 0.05 vs. the N‐BMSCs group.

Observation using an inverted microscope showed that BMSCs at passage 3 exhibited spindle‐shaped morphology and grew as adherent monolayers (Figure 2A). Moreover, no changes were observed in cell morphology under hypoxic conditions for 48 h (Figure 2B). Flow cytometry analysis demonstrated that 99.3% of the cells expressed CD 90 and 99.3% expressed CD29, all of which are surface markers of bone marrow‐derived mesenchymal stem cells, while only a small percentage of cells expressed CD 11b and CD 45 (Figure 2C). These results confirmed that the purity of BMSCs was higher than 99%, appropriate for the subsequent experiments. Cells were then exposed to normoxia (21% O_2_) or hypoxia (3% O_2_) for 48 h, and the CCK‐8 assay showed that cells cultured in hypoxia for 48 h did not exhibit higher proliferation (Figure 2D).

Subsequently, the apoptosis rate was measured by flow cytometry assay using Annexin V/PI labelling, and the results indicated that the apoptotic rate in BMSCs cultured under hypoxic conditions did not increase, and there was no significant statistical difference between N‐BMSCs and HP‐BMSCs (Figure 2E,F). Finally, the Transwell assay was conducted to evaluate the cell migratory capacity under hypoxic conditions. The results revealed that HP significantly increased the migration of BMSCs (Figure 2G,H; *p* < 0.05). These findings suggested that non‐fatal hypoxic preconditioning for 48 h enhanced the migratory ability of BMSCs, which would make HP‐BMSCs more favourable for engraftment than N‐BMSCs.

### 3.2 Physiological parameters at baseline and during the CPR modelling

In the current study, there were 72 male rats were included who achieved ROSC. These rats were randomly selected and assigned to four groups, each consisting of 18 rats. Each group was further divided into three subgroups based on the sample collection time points (6, 12 and 24 h after ROSC, *n* = 6 per subgroup). Additionally, 15 rats were selected as the sham group, with five rats in each subgroup.

There were no statistically significant differences in the physiological parameters among the five groups. Moreover, the time from asphyxia to cardiac arrest, adrenaline dosage, time from CPR to achieve ROSC and duration of hypoxia was similar in all groups, with no statistically significant differences (Table 1).

**TABLE 1:** Baseline and modelling parameters.

Physiological parameters	Sham			CPR			CPR + PBS			CPR + N‐BMSCs			CPR + HP‐BMSCs		
	6 h	12 h	24 h	6 h	12 h	24 h	6 h	12 h	24 h	6 h	12 h	24 h	6 h	12 h	24 h
Body weight (g)	221.0 ± 5.9	231.1 ± 11.2	221.3 ± 18.8	218.5 ± 12.3	212.3 ± 10.6	219.1 ± 16.9	223.2 ± 12.0	232.7 ± 10.2	233.2 ± 12.8	230.6 ± 13.7	223.9 ± 14.8	224.0 ± 13.5	222.3 ± 12.4	230.8 ± 16.2	230.5 ± 11.3
Rectal temperature (°C)	36.9 ± 0.2	37.0 ± 0.3	36.9 ± 0.4	36.8 ± 0.2	36.8 ± 0.3	37.0 ± 0.3	36.9 ± 0.3	37.0 ± 0.2	37.0 ± 0.4	36.9 ± 0.3	36.9 ± 0.4	36.9 ± 0.3	37.0 ± 0.2	37.0 ± 0.3	36.7 ± 0.2
Heart rates (beats/min)	361.5 ± 26.2	373 ± 27.4	362.5 ± 29.1	367.7 ± 17.9	348.2 ± 19.2	381.3 ± 20.3	364.7 ± 12.0	368 ± 26.2	381.3 ± 38.8	361.5 ± 40.8	355.2 ± 25.9	365.0 ± 26.4	366.5 ± 30.0	373.5 ± 30.9	377.5 ± 22.0
Mean arterial pressure (mmHg)	113.3 ± 10.7	112.2 ± 12.3	114.3 ± 10.2	116.5 ± 8.3	119.3 ± 8.6	113.8 ± 8.8	118.7 ± 5.6	117.8 ± 8.7	118.4 ± 7.6	108.7 ± 9.5	108.5 ± 7.2	112.3 ± 4.4	115.0 ± 2.3	114.3 ± 10.2	114.3 ± 6.9
Time from asphyxia to cardiac arrest (s)	N/A	N/A	N/A	224.3 ± 19.2	226.5 ± 21.2	214.3 ± 13.3	235.3 ± 19.2	219.7 ± 21.7	232.2 ± 12.6	233.8 ± 25.2	228.2 ± 24.0	240.5 ± 18.2	236.5 ± 18.9	242.2 ± 20.0	223.8 ± 19.8
Adrenaline dosage (μg)	N/A	N/A	N/A	10.9 ± 0.6	10.6 ± 0.5	11.0 ± 0.8	11.2 ± 0.6	11.6 ± 0.5	11.7 ± 0.6	11.5 ± 0.7	11.2 ± 0.7	11.2 ± 0.7	11.1 ± 0.6	11.5 ± 0.8	11.5 ± 0.6
Time from CPR to achieve ROSC (s)	N/A	N/A	N/A	91.3 ± 13.8	94.7 ± 18.2	88.7 ± 15.1	85.5 ± 13.9	97.3 ± 16.9	93.8 ± 13.4	86.0 ± 18.4	87.8 ± 22.3	90.2 ± 18.6	88.7 ± 20.2	86.8 ± 17.5	85.7 ± 16.6
Duration of hypoxia (s)	N/A	N/A	N/A	795.7 ± 26.2	801.2 ± 24.3	783.0 ± 7.4	800.8 ± 30.5	797.0 ± 31.7	806.0 ± 22.8	799.8 ± 36.8	796.0 ± 38.9	810.7 ± 28.3	805.2 ± 28.9	809.0 ± 36.4	796.7 ± 27.2

*Note*: Data are expressed as means ± SD.

Abbreviations: CPR, cardiopulmonary resuscitation; ROSC, the return of spontaneous circulation.

### 3.3 Luciferase‐base live imaging at 6 h, 12 h and 24 h after BMSCs transplantation

**FIGURE 3:**
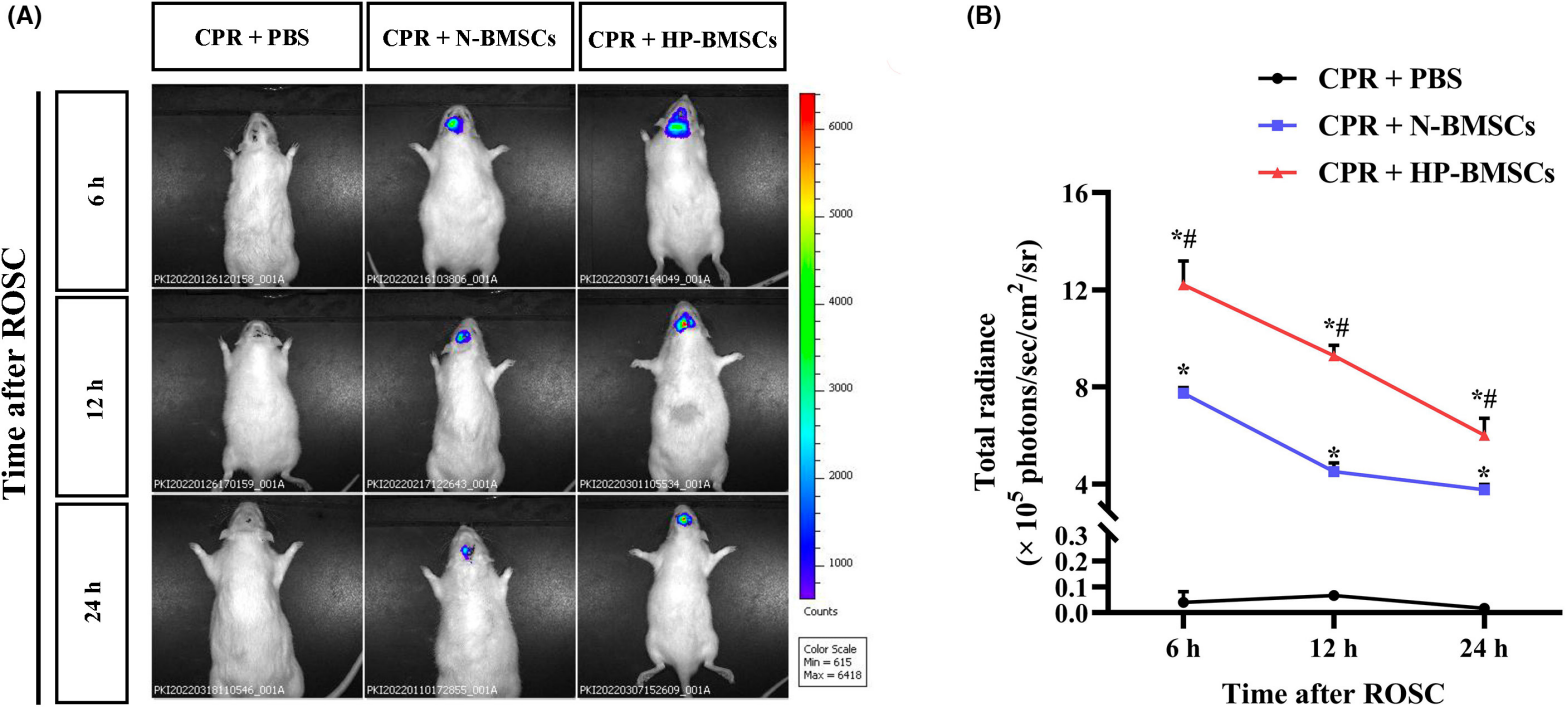
In vivo cell tracking and evaluation of the effects of hypoxic condition on the survival of engrafted bone marrow‐derived mesenchymal stem cells (BMSCs) after intracerebroventricular injection. (A) Representative bioluminescence images (BLI) show that hypoxic preconditioning enhanced BMSCs survival at 6 h, 12 h and 24 h post‐transplantation compared to the normoxic cultured cells. (B) The bioluminescence from the regions of interest (ROI) in rat heads, and the results are presented as the total radiance ± SD (*n* = 3). *p < 0.05 vs. the CPR + PBS group. ^#^ *p* < 0.05 vs. the CPR + normal‐cultured BMSCs (N‐BMSCs) group.

In vivo imaging system was used to measure and quantify bioluminescence signals at time points following intracerebroventricular treatment to better track HP's effects on BMSC survival and migration in the rat CPR model (Figure 3A). The survival and distribution of BMSCs were indicated by the intensity and ranges of the luciferase signals, respectively. The lateral ventricle of the negative control group received the same injection of 15 μL PBS. According to representative bioluminescence imaging (BLI) and quantitative analysis, the CPR + PBS group exhibited no or low bioluminescence signals. The dispersion of BMSCs in the brain was observed 6 h after implantation. During 24 h, the bioluminescence's intensity progressively reduced. Compared to the control group, the total radiance in both BMSC‐transplanted groups was noticeably higher at each time point (*p* < 0.05). Furthermore, hypoxic preconditioning for 48 h facilitated survival and migration of transplanted BMSCs, compared with the BMSCs cultured in normoxia (Figure 3B). The above differences were statistically significant (*p* < 0.05).

### 3.4 Hypoxic preconditioning enhanced the effects of BMSCs on alleviating cerebral injury

**FIGURE 4:**
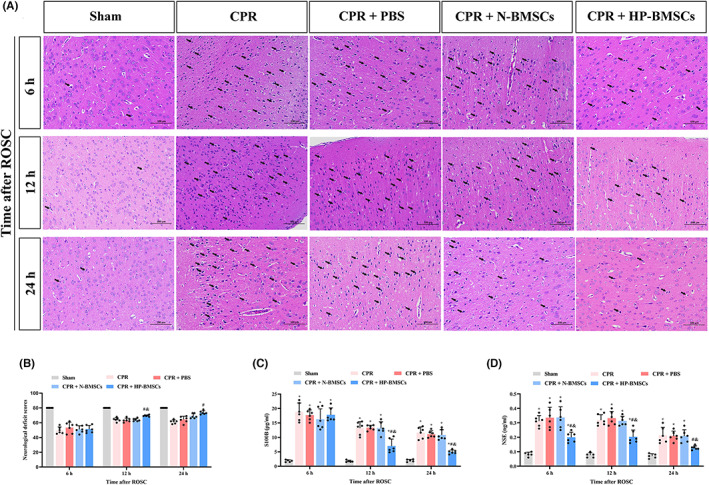
Hypoxic preconditioned bone marrow‐derived mesenchymal stem cells (HP‐BMSCs) attenuated neurological damage following cardiac arrest and cardiopulmonary resuscitation in rats. (A) HP‐BMSCs ameliorated the cerebral cortex pathological injury in rats with global cerebral ischaemia–reperfusion. Black arrows in representative images indicate damaged cells and mainly manifested as vacuolated cytoplasm with deeply stained nuclei. Scale bar = 100 μm (*n* = 5–6). (B) Neurological deficit scores showed HP‐BMSCs improved neurologic function recovery after resuscitation (*n* = 5–6). (C, D) The serum S100B and neuron‐specific enolase (NSE) levels were reduced with intracerebroventricular HP‐BMSCs injection (*n* = 5–6). All data are presented as means ± SD. *p < 0.05 vs. the sham group. ^#^ *p* < 0.05 vs. the CPR + PBS group. ^&^ *p* < 0.05 vs. the CPR + normal‐cultured BMSCs (N‐BMSCs) group.

HE staining was used to observe the pathology of the cerebral cortex. As shown in Figure 4A, the cerebral cortex neurons were roughly normal in the sham group, with a stained nucleus and uniform cytoplasm. Instead, the brains from the CPR and CPR + PBS groups had several deeply stained nuclei and pyknoti changes with vacuolated cytoplasm. Even though the CPR + HP‐BMSCs group had CA/CPR damage, the cell morphology responded well to the HP‐BMSCs treatment at 12 and 24 h after transplantation. According to the results, HP‐BMSCs engraftment decreased the abnormal cortical cells earlier. Twenty‐four hours after injection, cell damage was attenuated in the CPR + N‐BMSCs group. Moreover, neurological deficit scores at 12 h post‐transplantation showed that the CPR + HP‐BMSCs group regained neurological function quicker than the CPR + PBS group (*p* < 0.05). Meanwhile, compared with the CPR + N‐BMSCs group, the CPR + HP‐BMSCs group had a significant neurological functional improvement (Figure 4B, *p* < 0.05). In addition, S100B and NSE were considered biomarkers of brain injury following CPR.^48^ Serum S100B and NSE levels were measured to evaluate brain damage following global cerebral ischaemia–reperfusion injury. At 12 and 24 h post‐resuscitation, ELISA reported that the blood S100B and NSE levels were lower in the CPR + HP‐BMSCs group compared to the CPR + PBS group (Figure 4C,D; *p* < 0.05). Similarly, lower serum S100B and NSE expressions were detected in CPR + HP‐BMSCs group than in CPR + N‐BMSCs at 12 h and 24 h after ROSC (*p* < 0.05). Also, NSE levels significantly reduced in CPR + HP‐BMSCs group at an earlier time point (*p* < 0.05). These findings indicated that HP‐BMSCs treatment might prevent the brain from ischaemia–reperfusion injury following resuscitation and is better than N‐BMSCs transplantation.

### 3.5 Hypoxic preconditioned BMSCs transplantation alleviated neuronal pyroptosis in the cerebral cortex after CPR

**FIGURE 5:**
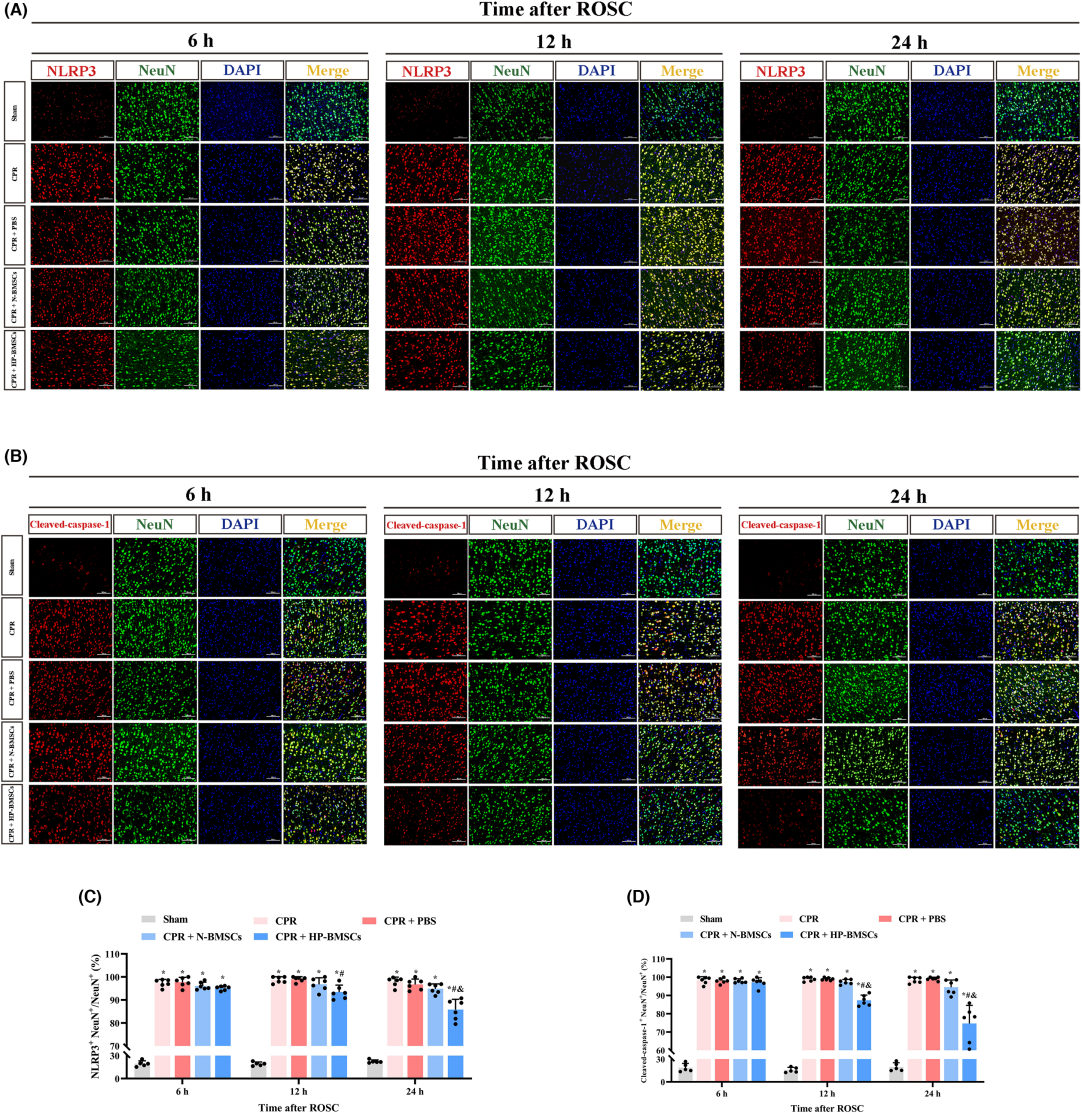
Hypoxic preconditioned bone marrow‐derived mesenchymal stem cells (HP‐BMSCs) ameliorated neuronal pyroptosis in cerebral cortex at various time points following resuscitation. (A, B) The expression of NLRP3 (red) and cleaved‐caspase‐1 (red) in neurons (NeuN, green) was examined by immunofluorescence double labelling and DAPI (blue) staining of the cell nuclei. Scale bar = 100 μm (*n* = 5–6). (C, D) Statistical comparison of NLRP3^+^ NeuN^+^/NeuN^+^ or cleaved‐caspase‐1^+^ NeuN^+^/NeuN^+^ levels among groups. The percentage of NLRP3^+^ NeuN^+^ cells within NeuN^+^ cells, and cleaved‐caspase‐1^+^ NeuN^+^/NeuN^+^ was similar. All data are expressed as the means ± SD. *p < 0.05 vs. the sham group. ^#^ *p* < 0.05 vs. the CPR + PBS group. ^&^ *p* < 0.05 vs. the normal‐cultured BMSCs (N‐BMSCs) group.

**FIGURE 6:**
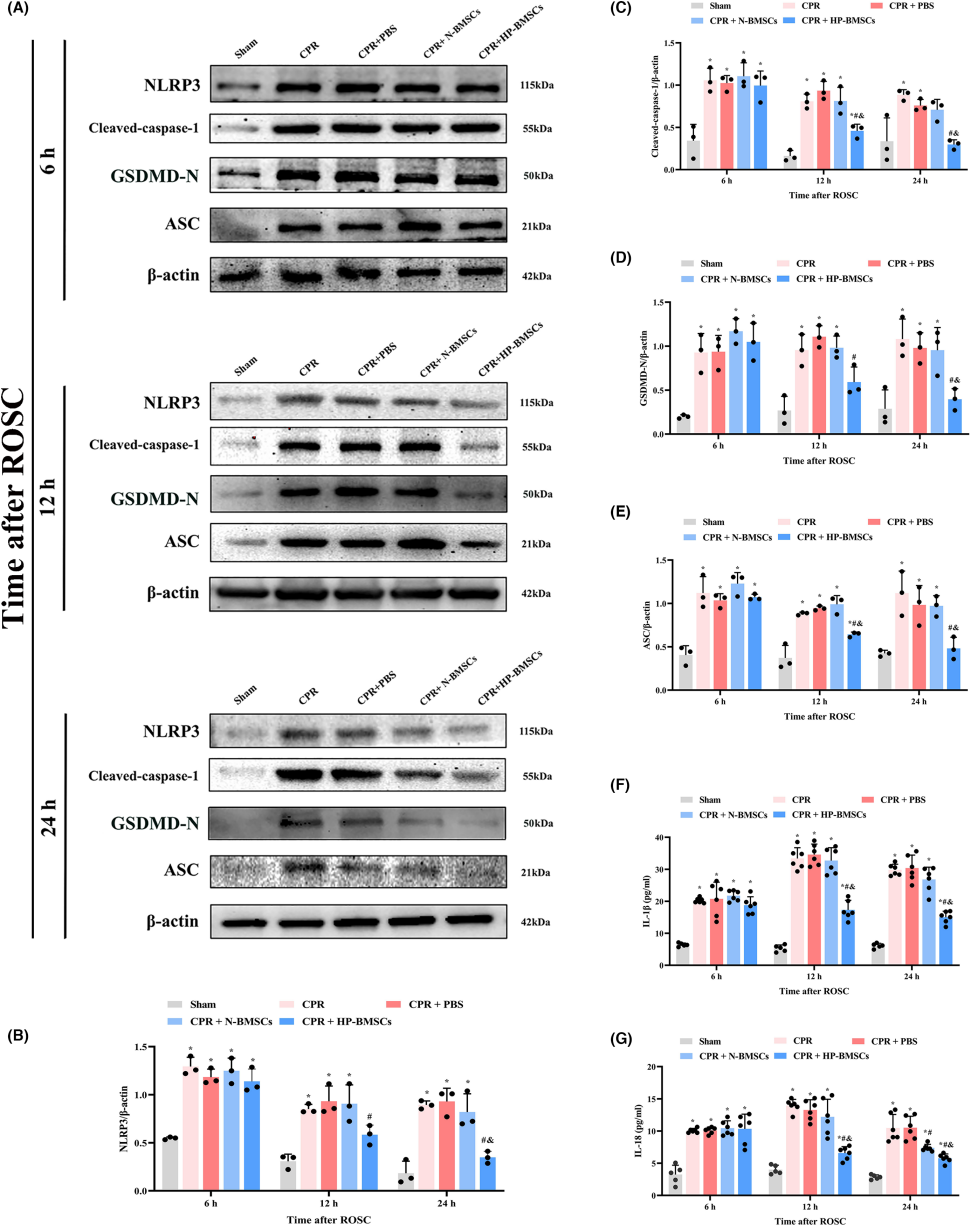
Hypoxic preconditioned bone marrow‐derived mesenchymal stem cells (HP‐BMSCs) reduced the expression of pyroptosis‐related molecules in the cortex after resuscitation. (A) Western blotting results showing expression of NLRP3, cleaved‐caspase‐1, GSDMD‐N and ASC in post‐resuscitation rats (*n* = 3). (B–E) Quantitative analysis of target proteins. (F, G) Expression level of IL‐1β and IL‐18 in the cortex after ROSC in the indicated groups (*n* = 5–6). All data are presented as the means ± SD. *p < 0.05 vs. the sham group. ^#^ *p* < 0.05 vs. the CPR + PBS group. ^&^ *p* < 0.05 vs. the CPR + normal‐cultured BMSCs (N‐BMSCs) group.

The expressions of NLRP3 and cleaved‐caspase‐1 were detected using double‐label immunofluorescence staining (Figure 5A,B) and immunofluorescent analysis performed on the cortical neurons after ROSC. After an 8‐min cardiac arrest, the proportions of NLRP3+ NeuN+/NeuN+ or cleaved‐caspase‐1+ NeuN+/NeuN+ increased significantly in the CPR group and CPR + PBS group (Figure 5C,D, *p* < 0.05), and both ratios were around 100%. The proportions of NLRP3^+^ NeuN^+^/NeuN^+^ or cleaved‐caspase‐1^+^ NeuN^+^/NeuN^+^ gradually decreased after HP‐BMSCs transplantation into the lateral cerebral ventricle at 12 h and 24 h post‐resuscitation compared with the CPR + PBS group (both *p* < 0.05). Moreover, at 24 h after ROSC, NLRP3+ NeuN+/NeuN+ or cleaved‐caspase‐1+ NeuN+/NeuN+ reduced although not significantly in the CPR + N‐BMSCs group compared to the CPR + PBS group (both *p* > 0.05). Nevertheless, compared to the CPR + N‐BMSCs group, the HP‐BMSCs group exhibited significant decreases in both parameters at 24 h post‐treatment (*p* < 0.05). Moreover, the expressions of NLRP3, ASC, cleaved‐caspase‐1 and GSDMD‐N in cortical tissues were detected by western blotting to study the role of NLRP3 inflammasome‐mediated pyroptosis in cerebral damage after ROSC (Figure 6A–E). As expected, the levels of pyroptosis‐related proteins in the cortex significantly increased 6–24 h after resuscitation compared to the sham group (both *p* < 0.05, complete data about WB are available in Figure S1). Also, global cerebral ischaemia–reperfusion damage increased the expression of inflammatory factors (IL‐1 and IL‐18) (Figure 6F,G). Moreover, BMSC transplantation partially restored the up‐regulation of pyroptosis‐related proteins and inflammatory mediators caused by ROSC, but not significantly (*p* < 0.05) compared to the CPR + PBS group. Notably, however, hypoxic preconditioning significantly strengthened the anti‐pyroptotic effects of BMSCs and produced these neuroprotective effects at 12 h post‐resuscitation earlier. These experiments suggested that HP‐BMSCs might prevent cortical neurons from NLRP3 inflammasome‐mediated pyroptosis under CA‐induced injury.

**FIGURE 7:**
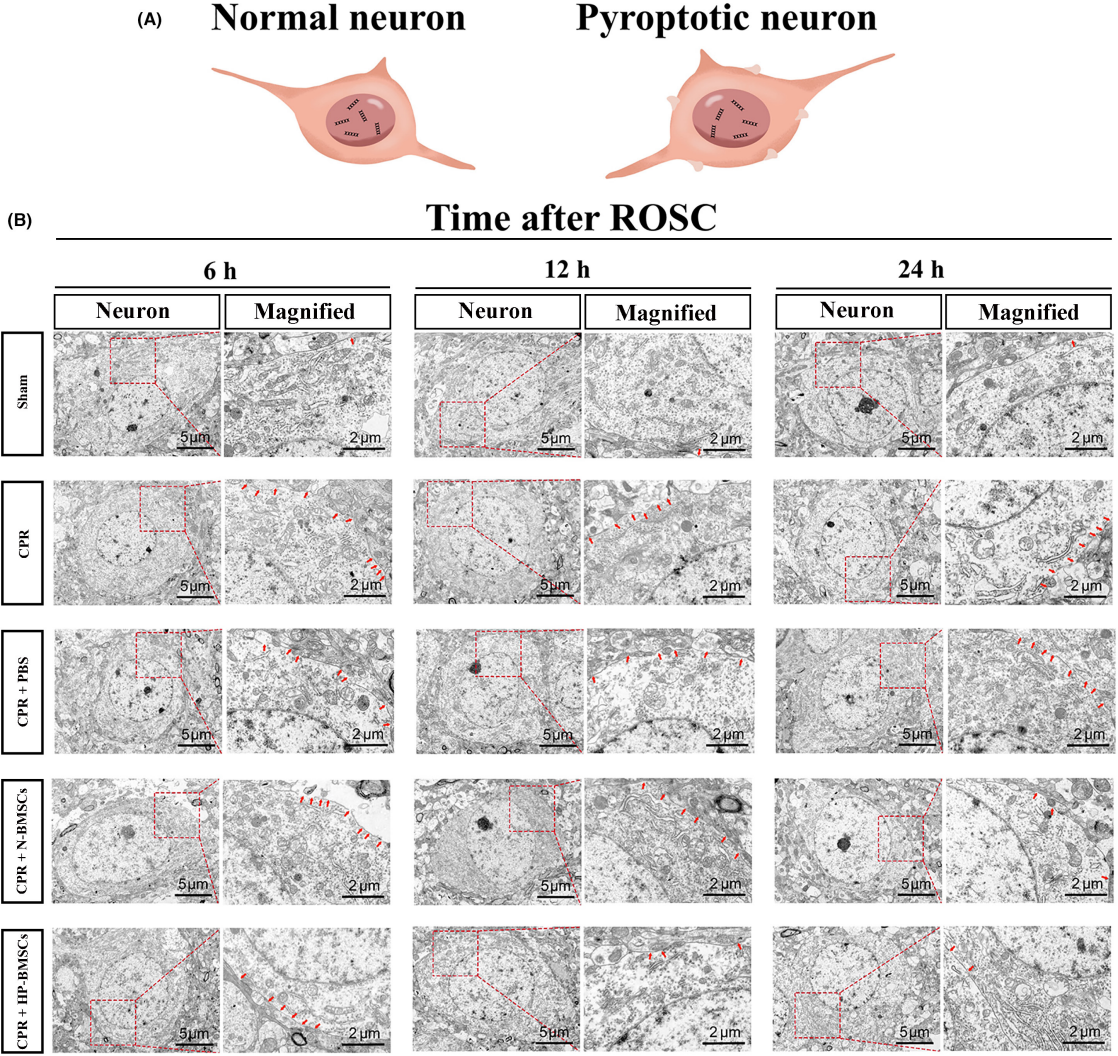
Representative pictures of electron microscopic examination demonstrated neuronal pyroptosis post‐resuscitation was alleviated by hypoxic pre‐conditioned bone marrow‐derived mesenchymal stem cells (HP‐BMSCs). (A) A schematic diagram showing the pyroptotic changes on the cell membrane. (B) HP‐BMSCs transplantation suppressed the cell membrane damage. Magnified images (Scale bar = 2 μm) of boxed areas in neurons (Scale bar = 5 μm) displaying the formation of holes in the membrane. The red arrows represent cell membrane pores (*n* = 3).

Membrane pore development is a common aspect of pyroptosis. We used TEM to examine the ultrastructural changes in cortical neurons at 6, 12 and 24 h after resuscitation (Figure 7A). After whole‐brain I/R injury and CA/CPR, pores emerge in the neurons' plasma membrane, and a membrane integrity breakdown (Figure 7B). Damaged neuronal boundaries were blurred and irregular, and mitochondrial disruption ensued. Nevertheless, according to previous findings, intracerebroventricular HP‐BMSCs injection significantly reduced this trend 12 h after resuscitation compared to N‐BMSCs injection. Our results also showed that HP‐BMSCs reduced NLRP3 inflammasome‐mediated cytokine release and thus protected the cerebral cortex from CPR‐induced whole‐brain injury.

### 3.6 Hypoxic preconditioned BMSCs treatment prevented neurological impairment by repressing activation of NF‐κB and MAPK signalling pathways

**FIGURE 8:**
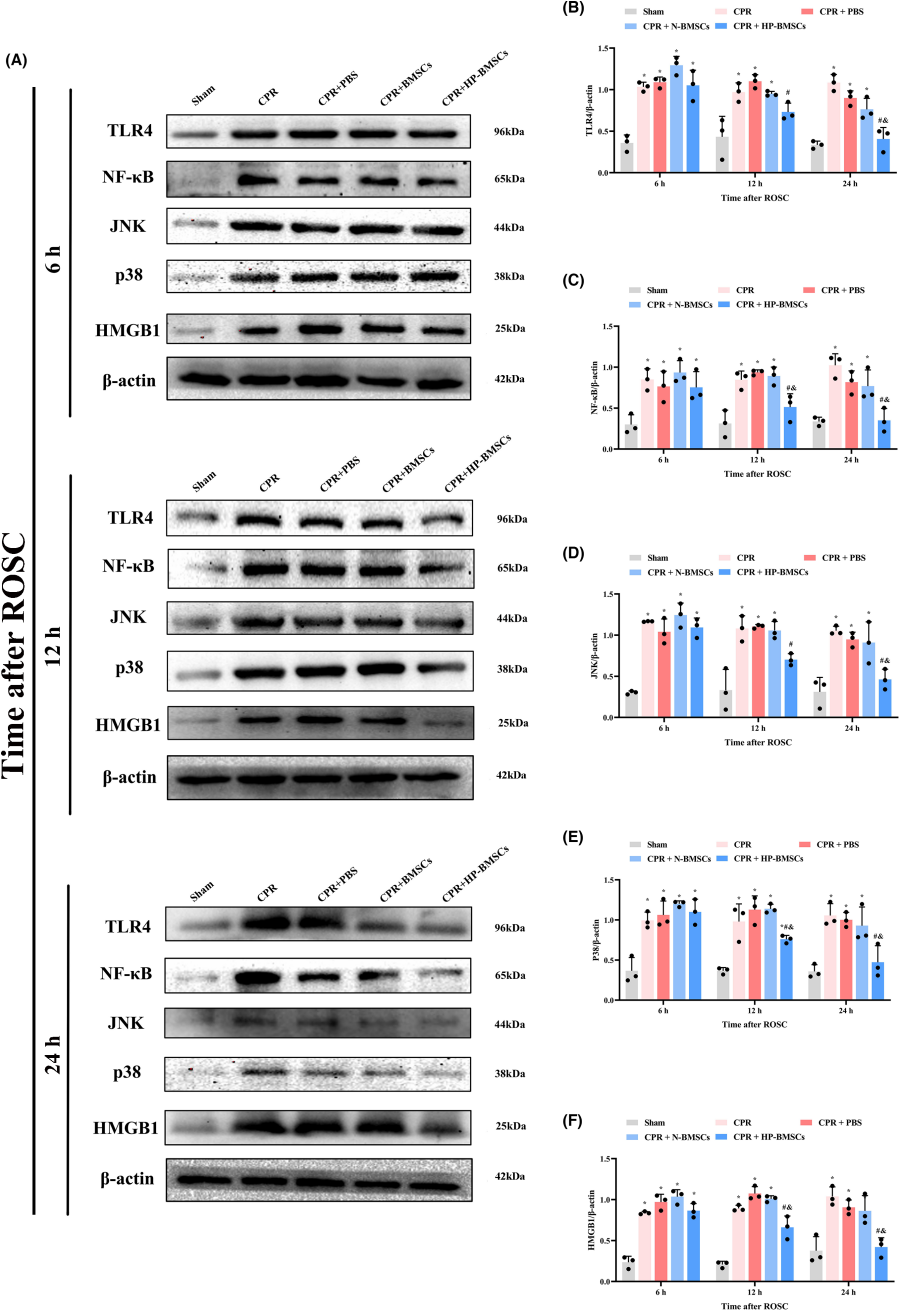
Effects of hypoxic preconditioned bone marrow‐derived mesenchymal stem cells (HP‐BMSCs) on the pyroptotic signalling pathway in cortical neurons after cardiac arrest. (A) Representative western blotting blots of TLR4, NF‐κB, JNK, p38 and HMGB1 proteins (*n* = 3). (B–F) Quantitative analysis of target proteins. All data are presented as the means ± SD. *p < 0.05 vs. the sham group. ^#^ *p* < 0.05 vs. the CPR + PBS group. ^&^ *p* < 0.05 vs. the CPR + normal‐cultured BMSCs (N‐BMSCs) group.

To better understand the impact of HP‐BMSCs on neurological dysfunction after ROSC, we investigated the protein expression levels of HMGB1, TLR4 and NF‐κB in the cortex (Figure 8A). Western blotting results confirmed that the proteins above significantly increased following CA compared to those in the control group (*p* < 0.05). Compared to the CPR or CPR + PBS groups, treatment with HP‐BMSCs reversed the CA‐induced alternation in HMGB1, TLR4 and NF‐B 24 h after resuscitation (*p* < 0.05). Moreover, compared to the CPR + N‐BMSCs group, the expression levels of these proteins began to decrease significantly after only 12 h following HP‐BMSCs implantation (Figure 8B,C,F, *p* < 0.05).

Subsequently, we evaluated the expressions of p38 MAPK and JNK in the cortex to better understand the impact of HP‐MSCs transplanted into the lateral ventricle on the MAPK signalling pathway of CA/CPR rats (Figure 8A). Our findings showed that p38 and JNK expression levels were considerably higher in CA rats compared to the sham group (*p* < 0.05). In contrast, intracerebroventricular injection of BMSCs resulted in a slight decrease in p38 and JNK expression, although this was not statistically significant (*p* > 0.05). Concurrently, this decline was significant for HP‐BMSCs treatment against the CPR + PBS group (*p* 0.05), and these proteins declined significantly at earlier time points (Figure 8D,E). It is important to note that the signalling pathway‐associated proteins followed the same pattern as the pyroptosis‐related proteins in response to HP‐BMSCs therapy (Replicate WBs are shown in Figure S1).

**FIGURE 9:**
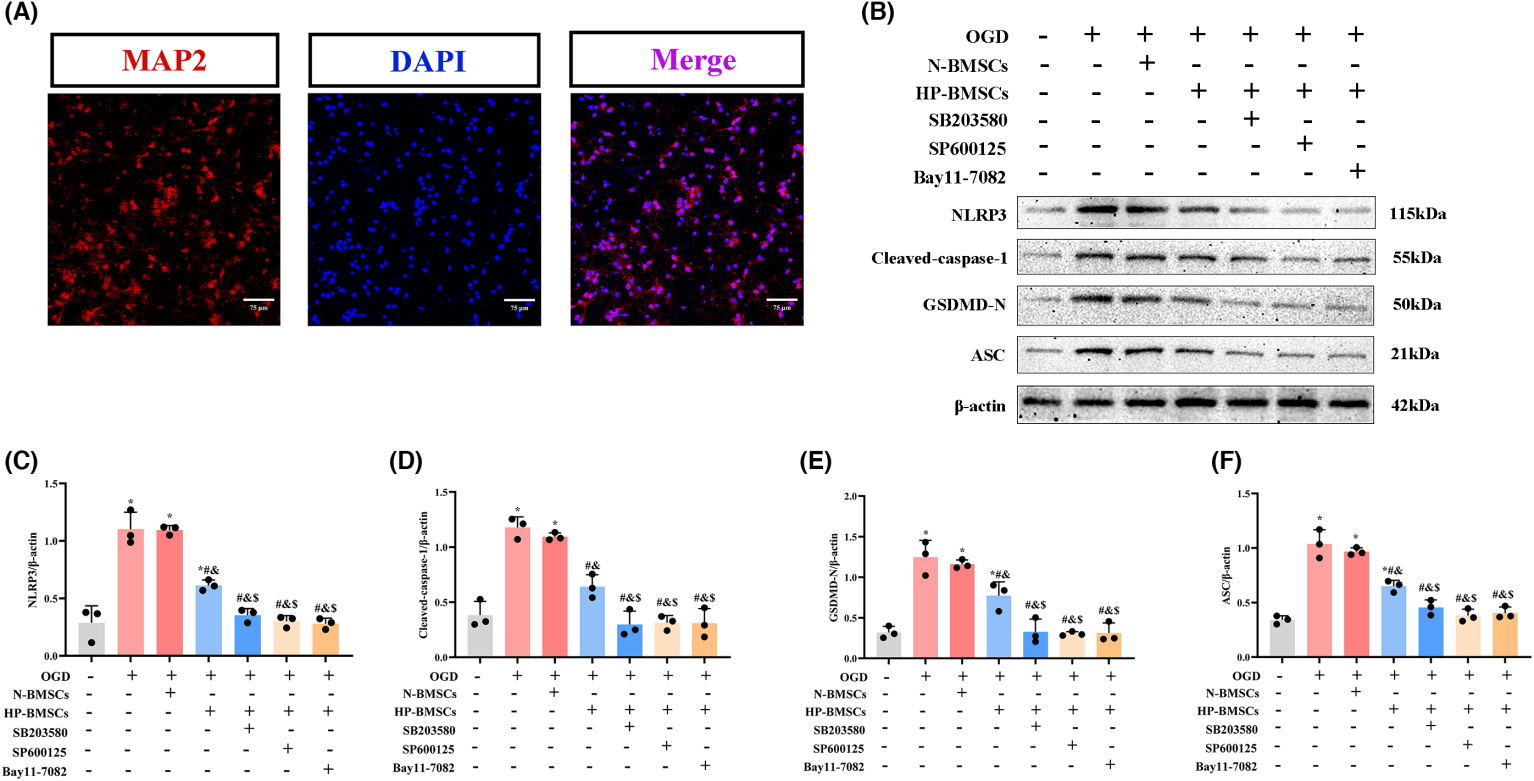
Anti‐pyroptotic effect of hypoxic preconditioned bone marrow‐derived mesenchymal stem cells (HP‐BMSCs) on OGD‐injured neurons involved the MAPK and NF‐κB signalling pathways. (A) Immunofluorescence indicated the presence of cell bodies, and neurites were labelled with anti‐MAP2 antibody (red), whereas nuclei were stained with DAPI (blue). Scale bar = 75 μm. (B) The protein expression levels of NLRP3, cleaved‐caspase‐1, GSDMD‐N and ASC in primary neurons in each group (*n* = 3). (C–F) Quantitative representation of the grayscale ratio of target protein/β‐Actin. All data are presented as the means ± SD. *p < 0.05 vs. the control group. ^#^ *p* < 0.05 vs. the OGD group. ^&^ *p* < 0.05 vs. the OGD + normal‐cultured BMSCs (N‐BMSCs) group. ^$^ *p* < 0.05 vs. the OGD + HP‐BMSCs group.

SB203580 (p38 MAPK inhibitor), SP600125 (JNK inhibitor) and Bay11‐7082 (NF‐B inhibitor) were added to the co‐culture system further to examine the pathway and mechanism of HP‐BMSCs on neurons. Cultured cells were stained positively for MAP2 (a neuronal marker, Figure 9A) in immunofluorescence labelling, showing that most cultured cells were neurons. As shown in Figure 9B, NLRP3, cleaved‐caspase‐1, GSDMD‐N and ASC were elevated in primary neurons after OGD compared to the control group (all *p* < 0.05). Pyroptosis‐associated proteins (NLRP3, cleaved‐caspase‐1, GSDMD‐N and ASC) in neurons reduced after co‐culture with N‐BMSCs; however, the trend was not apparent (all *p* > 0.05). In neurons co‐cultured with HP‐BMSCs, levels of pyroptosis‐associated proteins were much lower than in the OGD + N‐BMSCs group. Meanwhile, when SB203580, SP600125 or Bay11‐7082 were added to the co‐culture system, pyroptosis‐related protein expression levels in neurons were decreased further when compared to the OGD + HP‐BMSCs group (all *p* < 0.05). These in vitro results suggested that MAPK and NF‐B are involved in the mechanism of HP‐BMSC‐lowering neuronal pyroptosis (Replicate WBs shown in Figure S2). Generally, our findings indicated that transplanting HP‐BMSCs reduced pyroptotic cell death in CA‐induced neurological damage by suppressing the NF‐B and MAPK signalling pathways.

## 4 DISCUSSION

**FIGURE 10:**
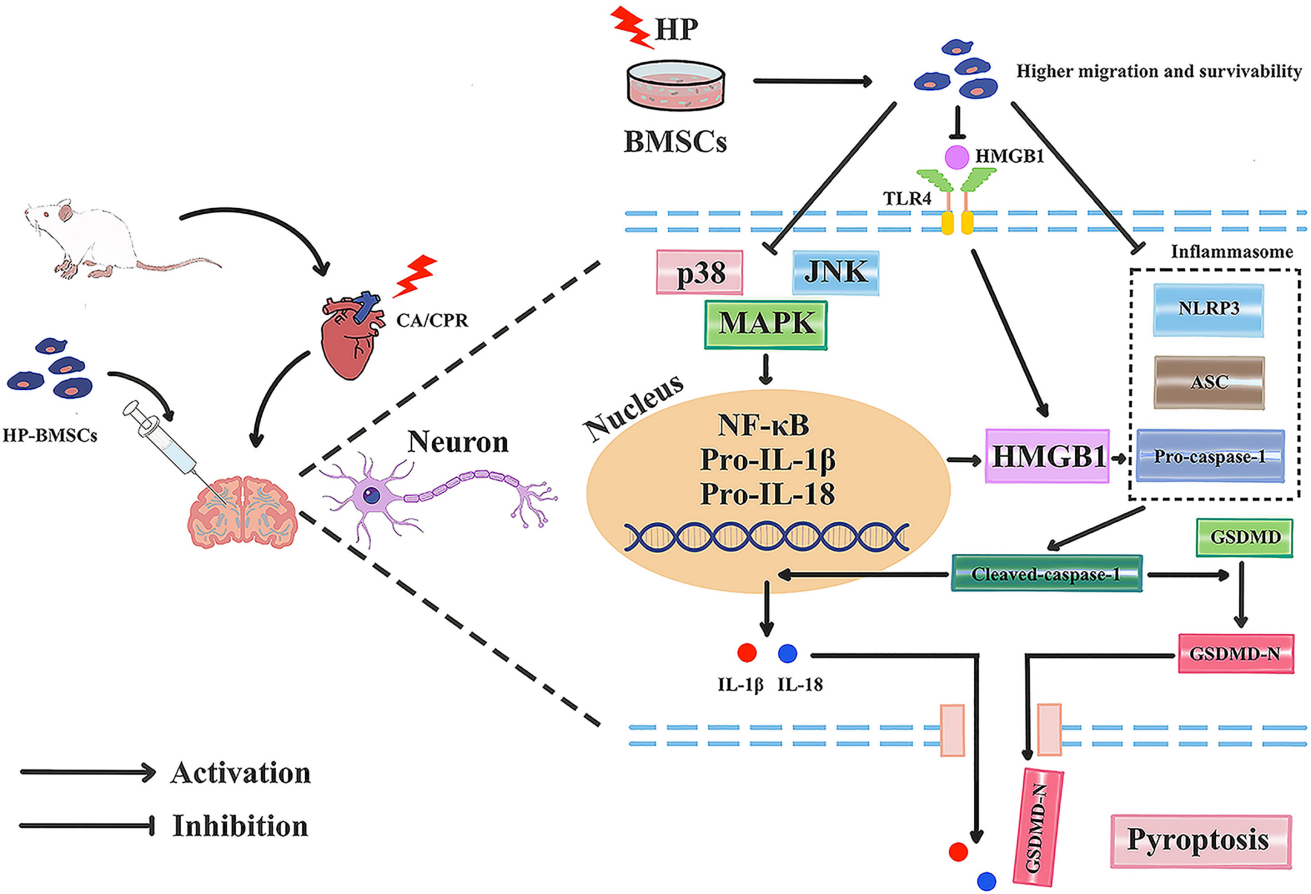
Schematic diagram illustrating the proposed mechanism by which hypoxic preconditioned bone marrow‐derived mesenchymal stem cells (HP‐BMSCs) alleviate neuronal pyroptosis via the NLRP3 inflammasome, MAPK and NF‐κB signalling pathways following cardiopulmonary resuscitation (CPR). The expression level of HMGB1 and TLR4 was upregulated following resuscitation, including MAPK pathway activation and pyroptosis initiation. This triggered the nuclear translocation of NF‐κB and increased HMGB1 leading to NLRP3 inflammasome formation. Subsequently, NLRP3 inflammasome activation promoted the cleavage and activation of pro‐caspase‐1, which contributed to elevation in IL‐1β, IL‐18 and GSDMD‐N levels, and induced pore formation on the membrane, causing pyroptotic cell death. Hypoxic preconditioning (HP) enhanced the tolerance capacity of BMSCs to local adverse microenvironment. HP‐BMSCs inhibited HMGB1 and TLR4 activation, which decreased MAPK and NF‐κB signals, as well as preventing the formation of NLRP3 inflammasome, which in turn ameliorated neuronal pyroptosis.

Cardiac arrest is a lethal disease with few treatment options and neurological dysfunction. Asphyxial CA is characterized by the loss of respiratory capacity, which causes rising hypoxemia and the cessation of heartbeats. The ratio of asphyxial CA has grown during the previous three decades,^49^ whereas the proportion of ventricular fibrillation CA has decreased.^50^ The aetiology of asphyxia CA includes several distinct causes, such as toxicology, upper airway obstruction and respiratory failure.^51^ Our study used the 8‐min asphyxial cardiac arrest and cardiopulmonary resuscitation model to mimic the pathophysiology of global cerebral ischaemia–reperfusion injury following resuscitation. We adopted an 8‐min no‐flow time based on earlier findings,^52^, ^53^, ^54^ which showed more prolonged periods of cerebral hypoxia and more severe brain injury than the standard 6‐min asphyxial CA rat model. There is limited research on the nervous system aetiology in rats following an asphyxia‐induced, 8‐min CA. We discovered that cerebral hypoxia might last up to 13–15 min in our model with significant brain lesions, which made resuscitation more difficult. Also, this implied that more effective treatments were needed, especially in light of the global ischemic brain's longer no‐flow duration. Our results show that (1) the CA/CPR model was successfully constructed with 8 min of no‐flow time; (2) asphyxia‐induced CA triggered neuronal pyroptosis in the cortex, mediated by the NLRP3 inflammasome; (3) HP‐BMSC intracerebroventricular transplantation significantly reduced neuronal pyroptosis, which improved brain tissue damage and the neurofunctional result after resuscitation; (4) HP‐BMSCs regulated the activation of HMGB1/TLR4/NF‐B and MAPK signalling pathways in response to CA, which was probably associated with the pathophysiological processes that HP‐BMSCs decrease pyroptosis and intracerebral inflammatory response to CA/CPR injury. This research used the CA/CPR model to evaluate global brain damage, which may contribute to I/R injury in organs other than the brain (heart, lung, liver and kidney), whose dysfunction may impede brain function. Given the specific pathophysiological processes of the CA/CPR model, we injected the BMSCs directly via intracerebroventricular administration through the brain stereo‐positioning instrument. The procedure eliminates the protective properties of BMSCs on these organs via systemic delivery post‐cardiac arrest. Furthermore, we show for the first time that HP‐BMSCs may protect against neuronal loss by inhibiting pyroptosis, reducing serum biomarkers for brain damage, and promoting neurological function recovery post‐transplantation in the CA/CPR model. This mechanism most probably involves HMGB1/TLR4/NF‐B and MAPK signalling pathways simultaneously in time (Figure 10).

Brain dysfunction caused by CA is the primary reason contributing to substantial morbidity and mortality following an initially successful CPR due to whole‐body I/R injury. According to statistics, the survival rate to discharge for those receiving out‐of‐hospital CPR is only 10.4%, and the survival rate with a favourable neurological prognosis is 8.2%.^55^ Due to the elevated impairment and mortality rates from CA in this population, further in‐depth study into post‐CA syndrome's physiological and pathophysiological processes is essential.^56^ Programmed cell death is one crucial pathogenic factor for post‐CA syndrome.^13^, ^57^, ^58^ Pyroptosis is a novel programmed cell death mechanism associated with inflammation, mediated by inflammatory caspase‐1 and caspase‐4/5/11.^59^ Cell swelling, plasma membrane pores and extravasation of cytosolic contents such as IL‐18, IL‐1 and lactate dehydrogenase (LDH) are significant pathogenic symptoms of pyroptosis.^60^, ^61^ Previous studies have shown that cells activate the inflammasome in response to various stimuli, such as the nucleotide‐binding oligomerization domain (NOD)‐like receptor (NLRP) family (NLRP1, NLRP3, NLRC4), which is absent in melanoma 2 (AIM2), or induce an intracellular signalling cascade via damage‐associated molecular patterns (DAMPs) and pathogen‐associated molecular patterns (PAMPs) bind.^60^, ^61^ NLRP3 inflammasome, including NLRP3, ASC and pro‐caspase‐1, are the most characteristic inflammasomes, which leads to auto‐cleavage and formation of active caspase‐1.^62^ Cleaved‐caspase‐1 processes the pro‐IL‐18 and pro‐IL‐1β into their activated forms, IL‐18 and IL‐1β. Meanwhile, cleaved‐caspase‐1 cleaves GSDMD, resulting in the reactive fragment GSDMD‐N and the auto‐inhibited fragment GSDMD‐C, which leads to pyroptotic cell death.^63^ Neuroinflammation is a crucial pathogenic factor in neurological diseases. Pyroptosis‐induced cell death has been reported in ischaemic brain tissue following cerebral infarction, triggering an inflammatory response and aggravating brain damage associated with elevated NLRP1 or NLRP3 inflammasome or caspase‐1 levels.^64^ Inflammasome is a multiprotein cytosolic complex participating in the innate immune response, inducing an inflammatory cascade. Several findings have shown that suppressing NLRP3 inflammasome‐mediated cell pyroptosis has neuroprotective and neurorestorative effects with cerebral I/R injury.^65^, ^66^ GSDMD ablation decreases pyroptosis in microglia, lowering infarction volume and enhancing neurological function in ischemic stroke patients.^67^ Similarly, in a rat CA/CPR model, anti‐pyroptotic therapy, MCC950 targets NLRP3 and Ac‐YVAD‐cmk targets caspase‐1, alleviates microglial pyroptosis and reduces neurological impairment.^13^ Notably, inhibition of pyroptosis in microglia or astrocytes and suppression of Drp1‐mediated inflammation improved mitochondrial morphology post‐resuscitation.^23^

Here, a CA model was developed to investigate the expressions of proteins linked to pyroptosis. Our data suggest that after ROSC, the cerebral cortex's expression levels of NLRP3, ASC, cleaved‐caspase‐1 and GSDMD increased with the number of membrane holes and inflammatory factor levels. The double‐label immunofluorescence labelling results confirmed the presence of NLRP3 or cleaved caspase‐1 in neurons; both expression ratios nearly approached 100% after 8 min of no‐flow time. Interestingly, few pyroptotic cells in the sham group might be partly related to repeated sevoflurane inhalation within 24 h.^68^, ^69^ In addition, after HP‐BMSCs transplantation to the lateral ventricles, the expressions of these proteins significantly decreased, and TEM images further confirmed the improved neuron membrane integrity. These results showed that HP‐BMSCs could suppress NLRP3 inflammasome‐mediated neuronal pyroptosis.

Many studies have reported the therapeutic potential of mesenchymal stem cells (MSCs) in treating brain injuries.^70^, ^71^ MSCs‐derived secretomes, including MSC‐derived extracellular vesicles (MSC‐EVs), conditioned medium (CM) or MSC‐derived exosomes (MSC‐EXOs), and MSCs themselves showed neuroprotective effects in various neurological disorders, such as stroke, multiple sclerosis, traumatic brain injury, spinal cord injury, Alzheimer's disease and Parkinson's disease.^70^, ^72^ They also migrated to the damaged area to differentiate into the terminal cells. MSCs affect immune regulation, paracrine effects, anti‐inflammatory roles, anti‐fibrotic actions and regulating non‐coding RNA.^73^ Our previous research suggested that MSCs transplantation relieved neuroinflammation and alleviated brain injury after CPR via regulating the inflammatory mediators.^34^ Our findings in this investigation indicated that MSCs decreased pyroptotic cell death and thus improved brain damage upon resuscitation. This result is consistent with recent research linking MSC therapy to neuronal pyroptosis after CA.^74^

Besides the therapeutic effects of MSCs on nerve function defects following ROSC, we investigated MSC's survival and migration in vivo. Previous studies report that numerous factors influence the survival of MSCs engraftment, including hypoxic microenvironment, insufficient blood supply, local inflammation, oxidative stress and nutritional deficiency.^75^ After grafting, MSCs depend on anaerobic glycolysis to maintain energy production under near‐anoxia conditions.^76^ Prolonged cellular starvation and depletion of energy reserves are likely in MSC apoptosis. PI3K/AKT/PTEN, p39/JNK, and p53 signalling pathways are reported to regulate ROS‐mediated apoptosis of MSCs post‐transplantation.^77^ Furthermore, intravenous infusion of MSCs has the potential to cause low homing efficiency and limited therapeutic efficacy.^78^ Limited engraftment, reduced paracrine action and low survival rate of the transplanted MSCs must be resolved in several ways.^79^ Researchers attempted to use genetic alteration, different cell delivery methods, pharmacological preconditioning and better incubation conditions to enhance the therapeutic effect of MSC.^80^, ^81^, ^82^, ^83^ Hypoxic preconditioning belongs to a type of modified culture method. Oxygen concentration in the natural niche of MSCs was between 1 and 5%,^84^ which was below the normal cultured levels (21%). Before intracerebroventricular injection, we assume cultivated BMSCs are maintained in a hypoxic environment (3%) for 48 h. Hypoxia might increase their transplantation efficiency and therapeutic impact. In vitro results indicated that MSCs did not alter cell morphology, depending on hypoxic culture condition (3%) for 48 h, stimulate MSCs proliferation or exhibit a pro‐apoptotic effect.

Our non‐lethal hypoxic culture increased cell migration, according to an in vitro Transwell experiment. Cell migration after injection is a crucial factor in the effectiveness of MSCs transplantation‐based therapy.^84^ Here, we quantitatively measured luciferase signals expressed in BMSCs to evaluate cell survival and migration in vivo. To the best of our knowledge, we reported, for the first time, the application of living imaging for BMSCs‐based therapy in the CPR model. Bioluminescent monitoring was characterized by a high signal‐to‐noise ratio and playing function only at the mammalian cell temperature of nearly 37°C.^85^ The generation of bioluminescence was significantly dependent on the host and grafted cells, and only live elicited a bioluminescent reaction.^86^ As a result, the bioluminescent system is a perfect technique for minimizing interference while tracking MSCs and observing their physiology. Subsequently, after 24 h of engraftment, we demonstrate that BMSC survival rapidly drops, whereas HP significantly improves declining cell vitality and migratory cell capacity.

Consequently, 48 h of HP are suitable for the transplanting of BMSCs. Nonetheless, no research has shown how HP‐BMSCs protect against post‐resuscitation neuronal pyroptosis. In this study, we found that HP‐BMSCs significantly diminished pyroptotic neurons, reduced the release of inflammatory mediators (IL‐1β and IL‐18) and improved cell membrane integrity compared to N‐BMSCs. Furthermore, we show time‐dependent variation in the expressions of proteins linked to pyroptosis (NLRP3, ASC, cleaved‐caspase‐1 and GSDMD‐N). Western blotting results and double labelling immunofluorescence indicate that HP can reduce the onset time of action after BMSCs administration, ultimately alleviating the brain tissue inflammatory injury after ROSC. Prior studies have shown that HP‐MSCs prevent microglial cells from death through pyroptosis and apoptosis by activating HIF‐1, a crucial transcriptional regulator associated with hypoxic conditions.^87^, ^88^ Another analysis revealed that ischemic–hypoxic preconditioned MSCs inhibit neuronal apoptosis and pyroptosis and protect mitochondrial function via miR‐181a signalling.^89^ However, more research is needed to examine the underlying processes of HP‐BMSCs' regulation of neuronal pyroptosis after CPR.

High mobility group box 1 is a ubiquitous nuclear protein, expressed highly in neurons, released from the cell nucleus, a characteristic feature of tissue damage.^90^, ^91^ As a cytokine or chemokine, HMGB1 has been proposed to have a function in triggering inflammation. The released HMGB1 is also recognized as a prototype DAMP.^92^ Moreover, HMGB1 transit from the nucleus to the extracellular environment was considered in another study, suggesting that HMGB1 secretion may be related to inflammasome complex activation.^93^ TLR4 is one of the HMGB1 receptors found outside of cells, along with TLR2, TLR4 and RAGE, and it is involved in neuroinflammation.^94^ Therefore, TLR4, a crucial pattern recognition receptor (PRR), can interact with DAMPs, contributing to the activation of NLRP3 inflammasome.^95^, ^96^ In addition, by triggering the p38 and/or JNK downstream signalling cascades, the binding of TLR4 to the ligand can activate the MAPK pathway, modulating the production of pro‐inflammatory cytokines in neuroinflammation.^97^ These signalling cascades are involved in the pyroptotic pathway.^98^ Again, the NF‐κB signalling transduction pathway can be activated following TLR4 activation.^99^ MAPK and NF‐κB signalling pathways are the classical cascades downstream of TLR4, and crosstalk exists between the two signalling pathways.^100^, ^101^ Moreover, it has been observed that TLR4 activation can increase HMGB1 levels via activating the NF‐B pathway.^102^ Also, NF‐κB, an essential transcription regulatory factor, is crucial in the pathogenesis of neuroinflammation.^103^ Research has demonstrated that MSCs inhibit cerebral cell apoptosis by downregulating the NF‐κB signalling pathway after brain damage.^104^, ^105^ In an I/R stroke model, TREM‐1 can activate CARD9/NF‐κB and NLRP3/caspase‐1, initiating microglia pyroptosis and releasing inflammatory factors. Another group has demonstrated that stroke‐induced white matter damage and microglial pyroptosis can be partially attenuated by suppressing the NF‐κB/NLRP3 signalling pathway.^106^ The latest study has indicated that the NF‐κB signalling pathway regulates pyroptosis in microglia post‐CA.^107^ Hence, the NF‐κB pathway was strongly connected to cell pyroptosis in nervous system diseases. Consist with studies mentioned above. According to our results, asphyxia‐induced global I/R damage increased the expression of the HMGB1, TLR4, p38, JNK and NF‐B. However, transplanting HP‐BMSCs restored these alterations and had a stronger anti‐inflammatory impact than N‐BMSCs. In addition, in the in vitro experiments, we further validated HP‐BMSCs could decrease OGD‐induced pyroptosis, and MAPK and NF‐κB signalling pathways might participate in this process. Hence, HP is a beneficial culture method to improve engraftment based on BMSCs. Moreover, HP‐BMSCs may be a useful strategy for preventing neurological damage following resuscitation.

Our study had several limitations. First, there are no clear guidelines for culture conditions in HP‐BMSCs, such as the appropriate period for hypoxic pretreatment and the optimal O_2_ concentration. Although our findings validated the neuroprotective benefits of HP‐BMSCs, additional study is needed to determine the best culture conditions. Second, our research did not address the molecular mechanism of HP leading to enhanced BMSC migration, which needs to be studied more in the future. Third, since many cell injections likely predispose to vascular embolization or local hematoma, we performed only single injections and only evaluated short‐term neurological function after ROSC.

Consequently, the effects of multiple HP‐BMSCs injections on long‐term neurological outcomes still require further study. Fourth, we did not detail the reasons for failing to achieve ROSC. Based on our observation, we found that the disappearance of cardiac electrical activity probably caused this during the no‐flow time. Finally, we did not perform the rescue experiment on HP‐BMSCs and signalling pathway activators. Despite being readily available, MAPK and NF‐κB activators have an unfavourable effect on the ROSC rate in our model, causing high animal attrition. Three‐time points were selected to observe the same trend for pyroptotic and signalling pathway‐related proteins, reducing experimental variations. In summary, the vast majority of current research studies are still at the experimental stage, indicating that it will take a significant amount of time before the translation of HP‐BMSCs into clinical applications can be achieved. Nevertheless, the use of HP‐BMSCs represents a promising novel approach for cerebral protection post‐resuscitation.

## 5 CONCLUSION

Our results showed that transplanted HP‐BMSCs alleviated CPR‐induced brain damage associated with the inhibition of NLRP3 inflammasome‐mediated pyroptosis. The neuroprotective effects are mediated by inhibition of the HMGB1/TLR4/NF‐κB and MAPK signalling pathways. These findings provide fresh evidence supporting the application of HP‐BMSCs post‐resuscitation.

## AUTHOR CONTRIBUTIONS

**Xiahong Tang:** Conceptualization (equal); methodology (lead); writing – original draft (lead); writing – review and editing (lead). **Jun Ke:** Conceptualization (equal); methodology (equal). **Falu Chen:** Methodology (supporting). **Qingming Lin:** Validation (equal). **Yan You:** Project administration (equal). **Nan Zheng:** Validation (equal). **Zheng Gong:** Data curation (supporting). **Xu Han:** Data curation (supporting). **Yangping Zhuang:** Validation (equal). **Feng Chen:** Conceptualization (lead).

## FUNDING INFORMATION

This study was supported by the Natural Science Fund of Fujian Province (no. 2020J011058), the Project of Fujian Provincial Hospital for High‐level Hospital Construction (no. 2020HSJJ12) and the Fujian Provincial Finance Department Special Fund (no. (2021) 848).

## CONFLICT OF INTEREST STATEMENT

The authors have no financial conflicts of interest.

## Supporting information


Appendix S1.

## Data Availability

All data and models in this study are included in the submitted article.
