# Supplementary material for: Hypoxic preconditioned mesenchymal stem cells ameliorate rat brain injury after cardiopulmonary resuscitation by suppressing neuronal pyroptosis
Source: J Cell Mol Med. 2023 May 29;27(13):1836–58. doi: 10.1111/jcmm.17782 (PMC10315812; doi:10.1111/jcmm.17782)
Supplement: Supplementary file 1 — Appendix S1. [file JCMM-27-1836-s001.docx]

Supplementary Material

Hypoxic preconditioned mesenchymal stem cells ameliorate rat brain injury after cardiopulmonary resuscitation by suppressing neuronal pyroptosis

Xiahong Tang^†^, Jun Ke^†^, Falu Chen, Qingming Lin, Nan Zheng, Yan You, Zheng Gong, Xu Han, Yangping Zhuang, Feng Chen^*^

*** Correspondence: Feng Chen: fjslcf@126.com**

# Supplementary Figure 1 The original figure of western blotting in brain tissues for three repeats

c
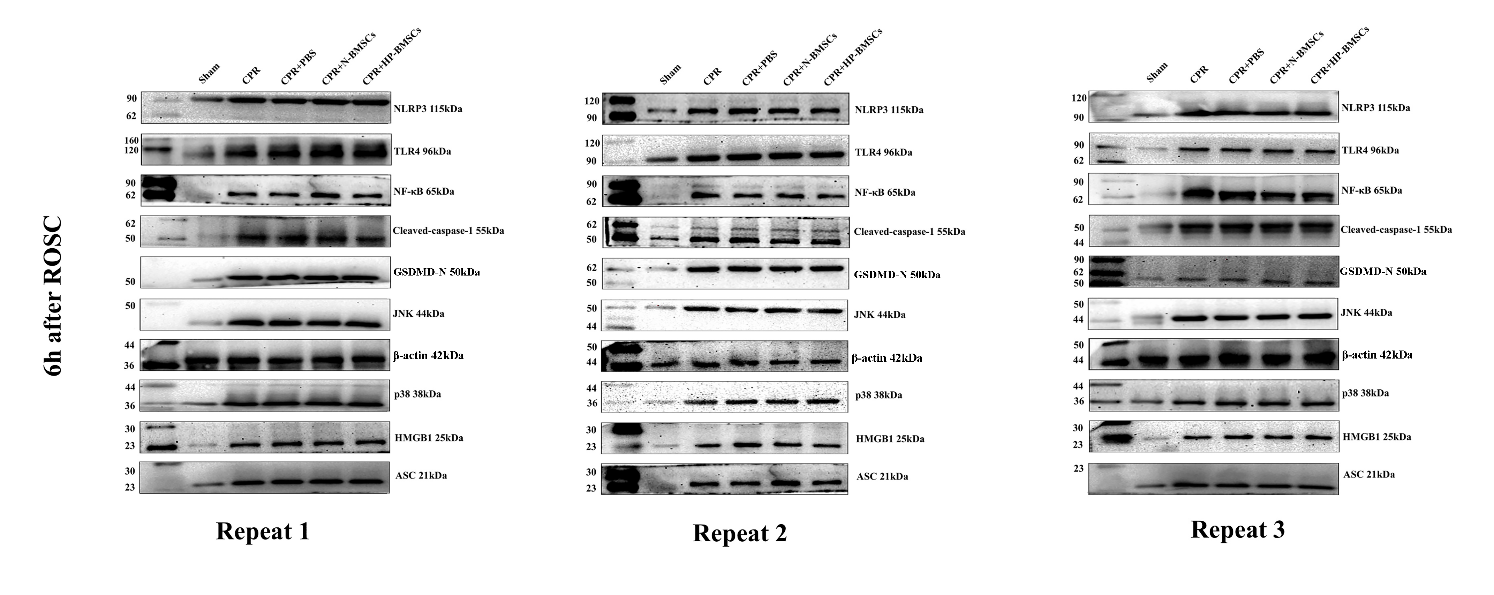


# The image for protein bands at 6h after ROSC


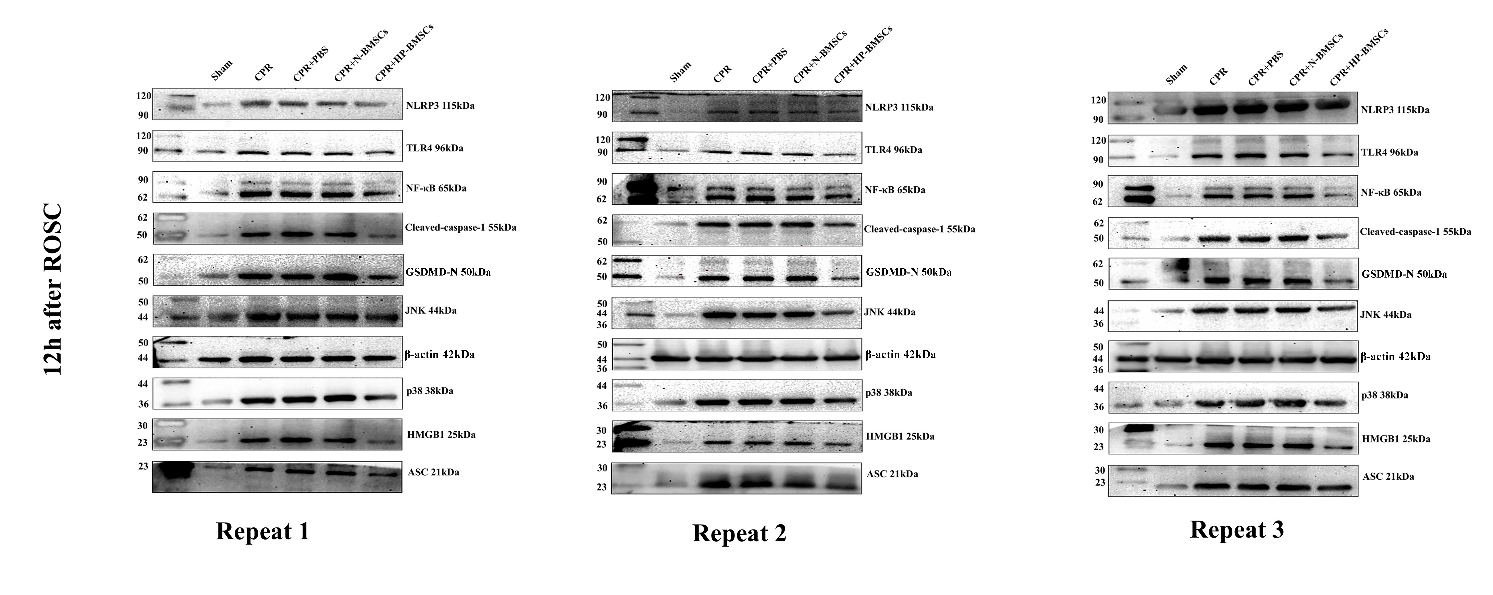


# The image for protein bands at 12h after ROSC

c
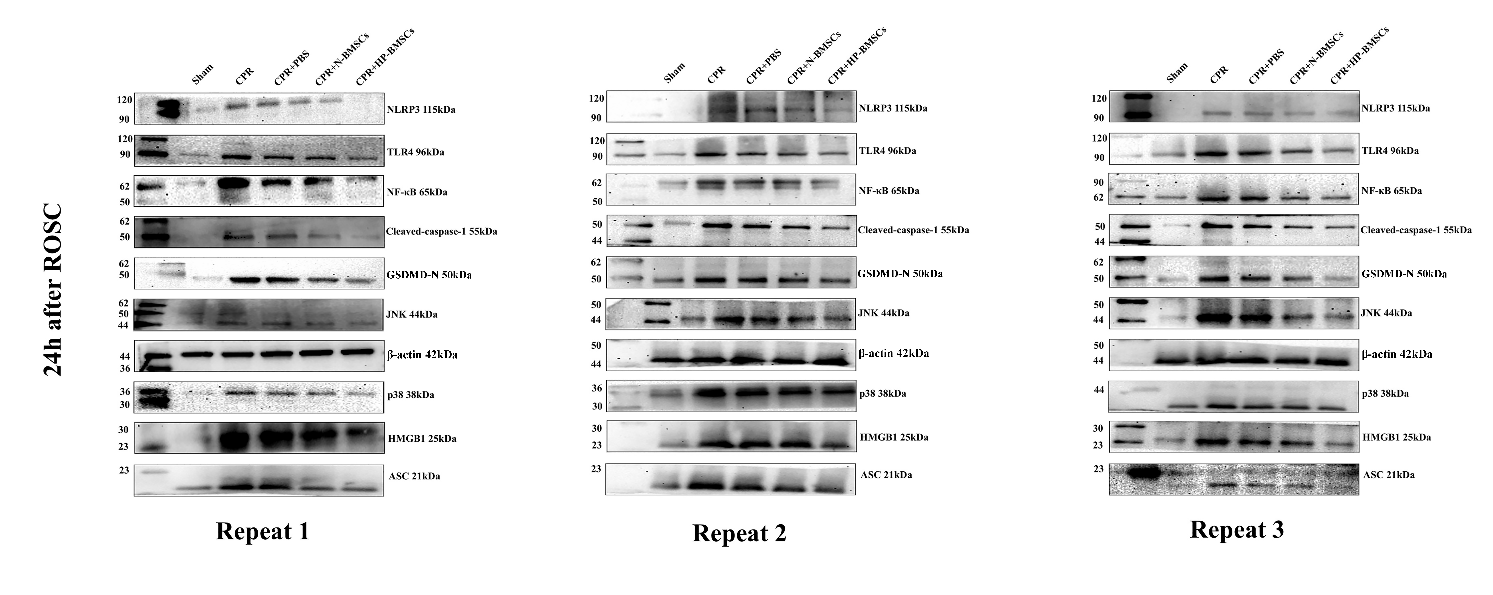


# The image for protein bands at 24h after ROSC

**Supplementary Figure 1.** The membrane was cut based on molecular weight of different proteins and the molecular marker was shown in each blot. Figure 1 shows the whole blot after cutting membrane for NLRP3 (115 kDa), TLR4 (96 kDa), NF-κB (65 kDa), cleaved-caspase-1 (55 kDa), GSDMD-N (50 kDa), JNK (44 kDa), β-actin (42 kDa), p38 (38 kDa), HMGB1(25 kDa) and ASC (21 kDa).

# Supplementary Figure 2 The original figure of western blotting in primary neurons for three repeats


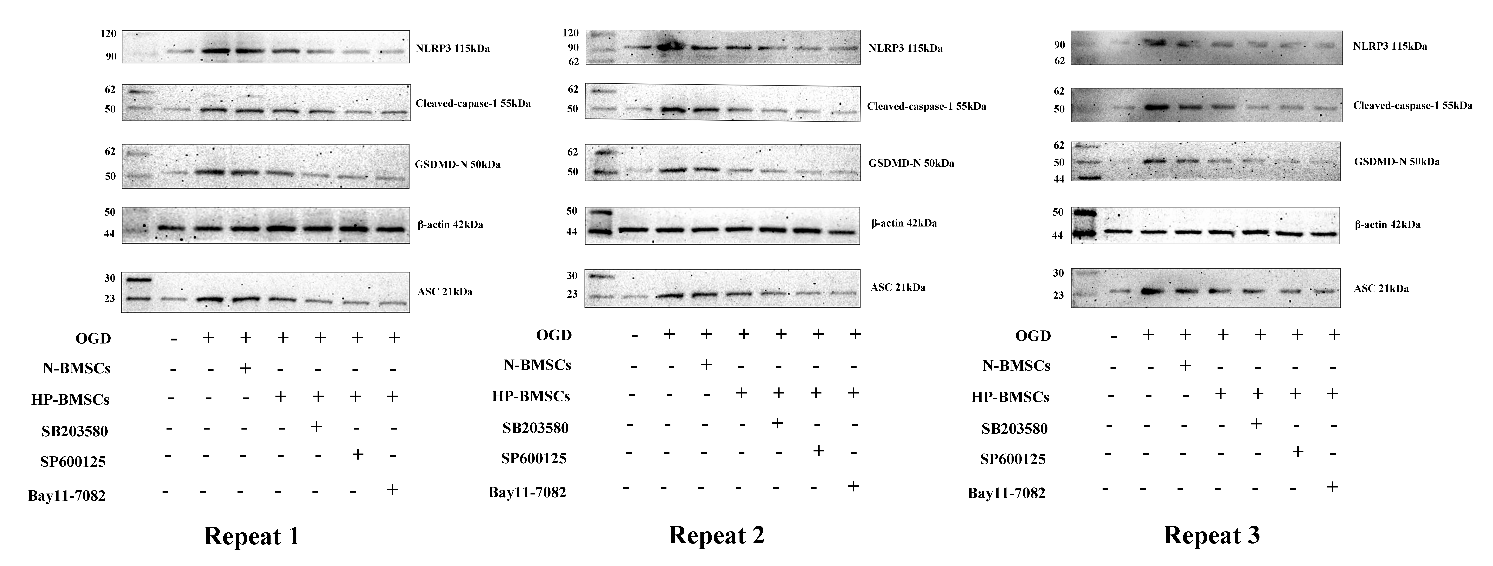


**Supplementary Figure 2.** The membrane was cut based on molecular weight of different proteins and the molecular marker was shown in each blot. Figure 2 shows the whole blot after cutting membrane for NLRP3 (115 kDa), cleaved-caspase-1 (55 kDa), GSDMD-N (50 kDa), β-actin (42 kDa) and ASC (21 kDa).
